# Notched implements made of scapulae (Bruszczewo-type tools)—A problem solved? Discovering cereal- and legume-threshing techniques in Early Bronze Age Europe through traceological analysis and residue studies

**DOI:** 10.1371/journal.pone.0308700

**Published:** 2024-09-13

**Authors:** Grzegorz Osipowicz, Justyna Orłowska, Emanuela Cristiani, Mariusz Bosiak, Lucy Kubiak-Martens, Janusz Czebreszuk, Daniel Makowiecki

**Affiliations:** 1 Institute of Archaeology, Nicolaus Copernicus University, Toruń, Poland; 2 Department of Oral and Maxillofacial Sciences, DANTE—Diet and Ancient Technology Laboratory, Sapienza University of Rome, Rome, Italy; 3 Department of Organic Chemistry, Faculty of Chemistry, Nicolaus Copernicus University, Toruń, Poland; 4 BIAX Biological Archaeology & Environmental Reconstruction, Zaandam, The Netherlands; 5 Faculty of Archaeology, Adam Mickiewicz University, Poznań, Poland; University of Haifa, Zinman Institute of Archaeology, ISRAEL

## Abstract

The studies presented in this paper constitute the first scientific attempt to interpret the manner whereby notched implements made of scapulae were made and used. These implements have been found at numerous European and non-European sites, usually dated to the Neolithic–Early Iron Age (predominantly the Early Bronze Age). Research has examined these products since the beginning of the 20th century, resulting in the development of several hypotheses regarding their functions. This paper presents the analysis results of 41 such artefacts from the early Bronze Age defensive settlement in Bruszczewo (central Poland). This is the largest collection of these products from a single site worldwide. The artefacts were subjected to multi-aspect traceological tests (both technological and functional) using optical, metallographic, and scanning electron microscopes. The residues identified on their surfaces were analysed using optical microscopy and scanning electron microscopy and energy dispersive X-rays spectroscopy. Moreover, the studies reported herein involved extensive experimental research. Consequently, the chain of operations followed in producing these tools was reconstructed and the use-wear traces present on their surfaces were classified; it was determined that these tools were most likely used for threshing cereals but might also have been used for threshing legumes. This is the first proof of threshing in central Europe in the Early Bronze Age and the first scientifically substantiated hypothesis regarding the function of these important artefacts.

## Introduction

Several collections of prehistoric artefacts include items with distinctive or mysterious forms, the purpose of which researchers have been preoccupied with for decades but, nevertheless, remains unknown. Some of these artefacts can be even considered ‘mass’ archaeological sources, as they occur at multiple sites in a large area and have a long chronology of utilisation. The classifications of these types of implements include, for instance, Mesolithic and Neolithic flint—so-called curved knives and microdenticulates [1–3], while products made of osseous raw materials include, among others, Magdalenian *bâtons percés* [4] and Mesolithic specimens [5, 6]. Moreover, the same category includes peculiar bone artefacts with concave or notched working edges made of animal scapulae, referred to in the literature as *Falzbeine* (groove bones) [7], toothed flax combs [8], notched implements made of scapulae [9], bone artefacts with serrated edges or scapular artefacts with serrated edges [10], and notched bone tools [11].

These tools have been found at numerous European prehistoric sites, primarily in collections dating back to the Early Bronze Age, in areas such as Germany, the Czech Republic, and Poland [12]. Furthermore, examples from contexts related to the Neolithic, Early Iron Age, and (to a lesser extent) pre-Roman Iron Age appear in these areas [9]. In Germany, they occur at sites such as Falkenwalde [13] and Lohberg [14]. However, additional information regarding other sites where they were unearthed can be found [15–18]. In the Czech Republic, their presence was recorded at, among others, Bánov, Hradisko, Charváty, and Praha Bubeneč [12, 19, 20]. In Poland, they were unearthed at various sites (except Bruszczewo), such as Jankowo [21], Kijewo [22], Biskupin [23], Borowo [24], Nowa Cerekwia [24–26], and Jędrychowice [27].

Outside Central Europe, similar notched artefacts have been identified in Estonia in layers of Late Bronze Age fortified settlements on the Island of Saaremaa (at the sites Asva, Kaali and Ridala) [10]. Interestingly, artefacts fundamentally identical in terms of morphology were also found at the multilayer wetland site Stanovoye 4, Russia [28]. They were termed ‘shoulder blades (“broad knife”) with sharpened edges’, with one of them radiocarbon-dated to the Early Holocene 9 879 ±50 BP [29]. Finds of this type from another Russian site located in Siberia, Blizhnie Elbany, date back to the 7^th^–6^th^ centuries BC [30]. Moreover, specimens of a similar form dating back to the Bronze Age and used at Oronte, Siria, dated back to the Bronze Age [31].

Therefore, the coverage and chronology of the artefacts in question are extremely broad. However, the most numerous collection of these implements (at the time of publication) originates from the fortified settlement in Bruszczewo, western Poland, dated to the Early Bronze Age [32, 33]. Dozens of products of this type (or fragments thereof) were unearthed at this site, most of which served as the subject of this research.

This research aimed to answer the questions of how the Bruszczewo artefacts were made and, primarily, their function, based on the results of traceological analysis and studies of the identified residues. Our findings can serve as a starting point for interpreting the purpose of similar objects at other European sites. To avoid unnecessary and unjustified generalisations (i.e. applying the findings presented herein to products from other collections without due diligence), as well as to emphasise the importance of the collection subject to the research conducted, the implements analysed in this research will be referred to as Bruszczewo-type tools.

### Previous studies on Bruszczewo-type tools`production techniques and function

To date, no extensive studies have examined the technology of the tools in question, and attempts to interpret their purpose are primarily speculations based on formal principles. Northe provided a detailed summary of investigations from the 20^th^ century regarding this tool type [9]. The most important of these interpretations and the suggestions proposed in the current century regarding the function of notched implements made of scapulae are presented here.

Initial attempts to explain the function of these artefacts were made in the early 20^th^ century by analysing selected Neolithic tools, when it was suggested that these tools were used for cutting meat [34] or sawing [35]. More detailed studies of this topic were conducted in the 1930s. Considering the morphology of (predominantly) Neolithic tools from Thuringia and the macroscopically observed use-wear damage on their surfaces, the function of the implements in question was associated with flax carding (detangling the fibres) [15]. This interpretation was later reiterated multiple times in connection with other finds dated back to the Neolithic [8], Late Bronze Age [36], and Early Iron Age [17].

However, some researchers have suggested different purposes for the scapulae tools in question. Rossius hypothesised that longitudinal, flat, and unnotched finds of this type dated to the Iron Age served as scrapers for working (tanning) hides [37]. A similar interpretation (skin deflesher) was proposed for an implement of this type found in northwestern Poland and dated to the Halstatt period [38]. Similarly, Neolithic products of this type from Thuringia have been identified as tanning implements [14]. Further, Walter and Mobes [18] associated these tools with the processing of animal materials. They proposed that these products were used in the butchering process, possibly for gut cleaning, rope-making, and tanning. This hypothesis was based on studies of Early Bronze Age finds of this type from the area of Thuringia and ethnographic analogies. The relationship between the tools, hide processing and, most likely, ‘cleaning, stretching and smoothing of tendons and guts’ is also backed by Northe [9]. Likewise, his interpretation draws extensively on ethnographic analogies, underscoring the fact that analogous implements were used by the Puebloan (for defurring) [39] and Innu people (for cleaning and softening hides) [40].

Tihelka drew a completely different conclusion regarding the likely function of the artefacts in question [19]. He believed that denticulated scapulae tools from the Early Bronze Age were used to make ceramics.

Products of this type were also considered plant-working tools, that is, implements used for refining plant fibres and making cords [41] and also used as sickles for grain harvesting [42, 43].

Some of the items of the discussed type found in Poland, dating back to the Lusatian culture period, were interpreted as musical instruments (ancient percussion instruments/musical rasp). This function was attributed, among others, to the scapulae artefacts unearthed in Jankowo [21] and Kijewo [22]. A similar use was suggested for a product of this type from the Oronte site, Siria, dated to the Bronze Age [31]. Per recent claims, these artefacts likely served multiple purposes [10].

Finally, exploring the results of traceological analyses and residue studies of practically analogous tools made of camel scapulae found at Boyo Paso 2 (late pre-Hispanic site, 1500–750 years BP; Sierras of Córdoba, Argentina) may be interesting. These products’ shapes are identical to those of Bruszczewo-type tools. The analysis results suggest that they were used for peeling the tubers of wild or domesticated *Oxalis* sp. [11].

As the above summary indicates, previous concepts the function of notched implements made of the scapulae are numerous and highly diverse.

### Bruszczewo site

**Fig 1:**
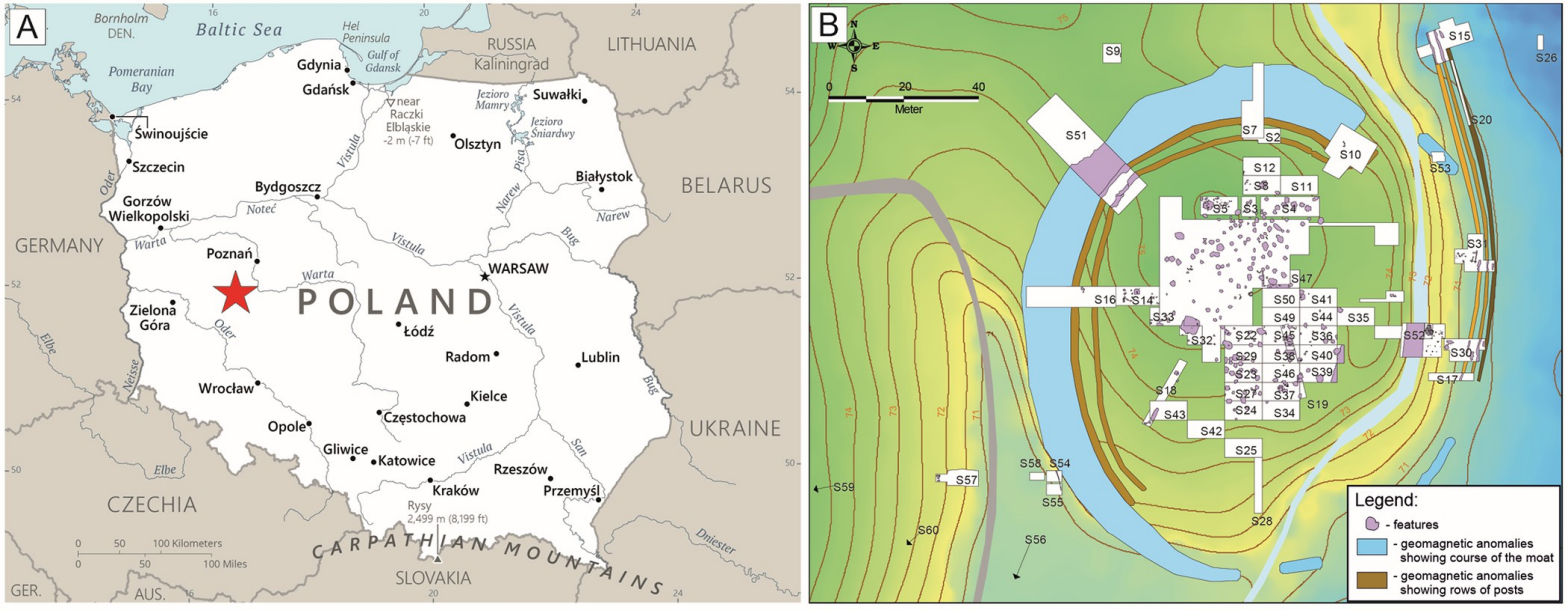
Location of Bruszczewo site in Poland (A) and plan of the site with research excavations marked (B). Data: (A) Background after The World Factbook 2021. Washington, DC: Central Intelligence Agency, 2021. https://www.cia.gov/the-world-factbook/countries/poland/map.

This Early Bronze Age settlement in Bruszczewo (Site 5) is located in Greater Poland (western Poland) on the border of three physiographic units, namely, the Sława Lakeland, Kościan Plain, and Krzywiń Lakeland (Fig 1A). It is located within a plane of sandur deposits composed of sand and gravel deposited by snow water on the undulating moraine upland of the North Polish glaciation’s Leszno stage. The site—located on a small peninsula protruding southeastward into the glacial trough of the Samica River—is isolated from sandur area (Fig 1B). Peat sediment fills the throughput and envelops the site on the southwestern side from the south, east, and northeast, limiting access from these directions. The peninsula reaches on average 75.6 m a.s.l. and covers an area of 1.5 hectares [44, 45].

The most important stage of prehistoric dwelling at the site is indicated by remains of a defensive settlement from the Bronze Age dated to the Únětice culture, covering about 500 years between 2100 and 1600 BC [46]. This settlement’s layout and spatial arrangements are known in detail owing to studies conducted at the site during 1964–1968 and 1995–2008 [33, 47–50]. Consequently, a central settlement was identified on the hill, and a lower settlement was identified in the peat zone (eastwards).

The central settlement was surrounded by a palisade (which, upon its establishment, doubled and subsequently tripled [46]) that outlined a circular area about 100 m in diameter (Fig 1B). Rows of stakes were set at approximately 2 m intervals, serving as the rampart’s face. The space between them was most likely filled with earth. On the site’s northern side, a moat up to 4 m deep and approximately 20 m wide was discovered that separated the settlement from the uplands. In this manner, a manmade island was established. Drilling and geomagnetic studies identified two entryways (gates) each leading inside the feature. Additionally, these studies suggest that the settlement was encircled by a third palisade situated between the rampart and dug-out moat [46, 51].

In the central settlements, several movable and immovable archaeological sources of various types were unearthed. Along with dwelling structures, perceived features included manufacturing structures related, for instance, to metallurgy. Moreover, bronze casting remains were identified, including multitudes of metal artefacts, thus confirming that the inhabitants were in contact with different European regions [32, 52].

Similarly, fortifications from the Early Bronze Age were found in the site’s peat zone. These comprise two parallel woven fences and a wooden wall, which would have formed the outer part of the defensive structure, and their origins date dendrochronologically to the early 18th century BC [53]. In the Early Bronze Age layers, highly abundant and well-preserved organic remains—such as grains, fruits, and numerous artefacts made of bone and antler—were unearthed. In this zone, a grave dated to the Early Bronze Age was discovered, specifically, the burial of an adult male lying on a woven willow mat [54, 55].

## Materials and methods

### Materials

**Fig 2:**
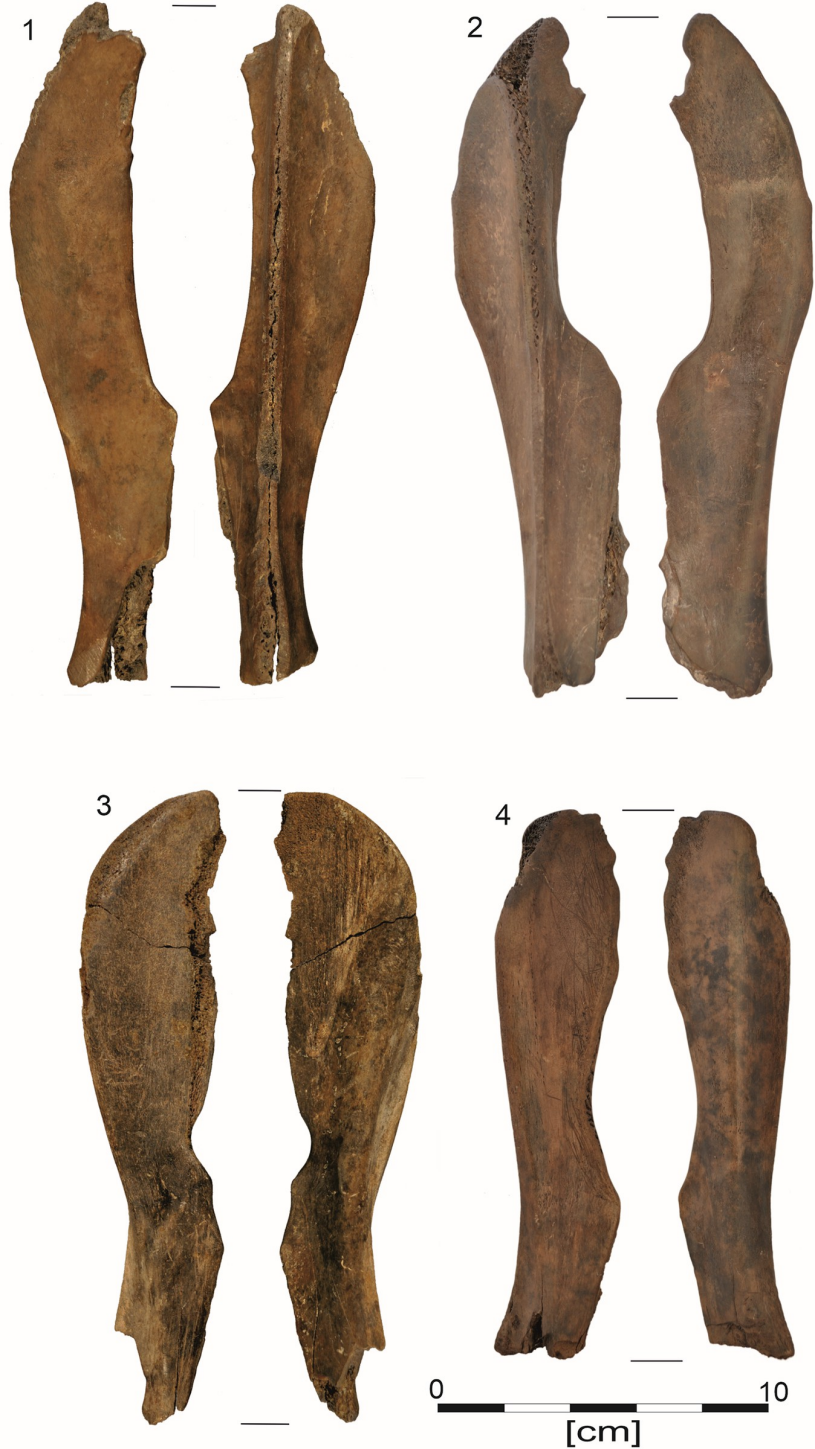
Examples of Bruszczewo-type tools.

**Fig 3:**
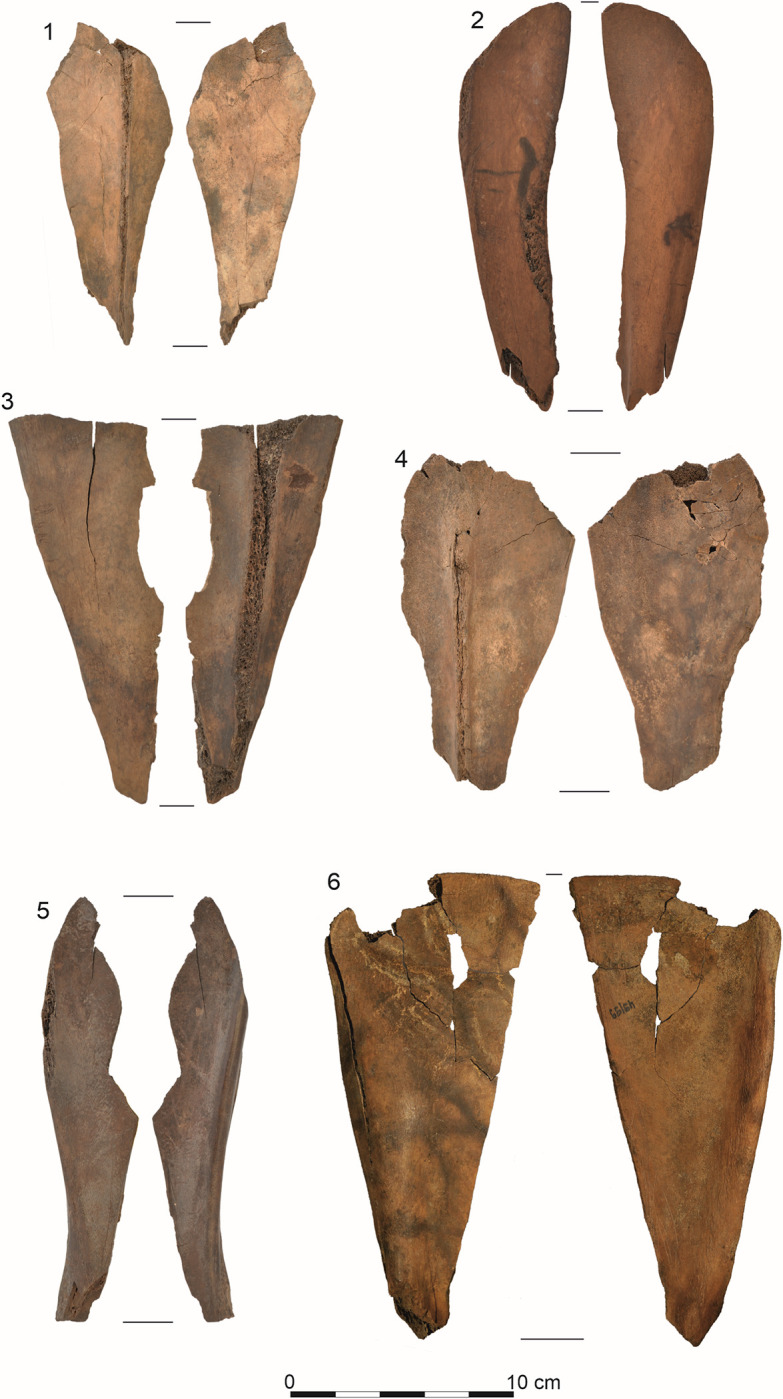
Examples of Bruszczewo-type tools (1–5) and a semi-product (6).

**Fig 4:**
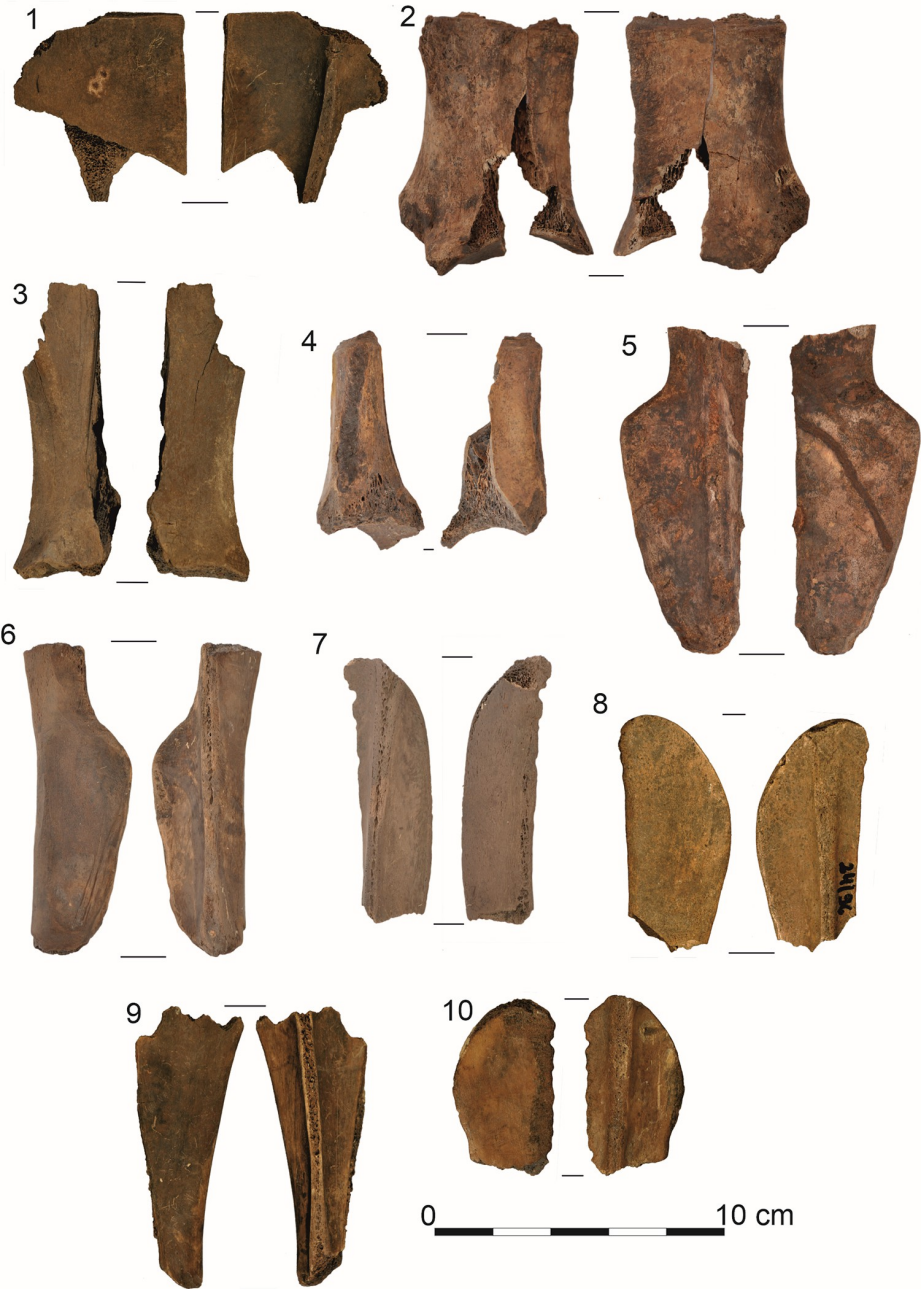
Examples of Bruszczewo-type tools preserved in fragments (1, 5–10) and waste from the production of tools of this type (2–4).

The subjects of this research were a collection of 41 osseous products from Site 5 in Bruszczewo, including 36 Bruszczewo-type tools, 13 of which are essentially preserved in their entirety (Figs 2: 1–4; 3: 1–5), while 24 are fragments of various sizes (Fig 4: 1, 5–10). The collection is complemented by a semi-product (unfinished tools) from the manufacturing of this form (Fig 3: 6) and three fragments of production refuse (Figs 2–4). All the covered artefacts were unearthed in the site’s peat zone.

A significant advantage of this collection is that various stages of the manufacturing process and different stages of use are represented. Consequently, detailed technological and functional studies—with a focus on reconstructing comprehensive biographies of the tools, all the way from choosing the raw material and crafting to being abandoned as waste—can be conducted. (S1 Table) presents the artefacts’ full profiles.

### Methods

The studies presented in this paper were conducted with the consent and co-authorship of Prof. J. Czebreszuk, the head of archaeological research at the site in Bruszczewo and the leader of the international research project dedicated to this site. With his consent, we received access to information regarding the field site and to artefacts that are the subject of the analysis (stored in the warehouses of the Faculty of Archaeology, Adam Mickiewicz University in Poznań and the Archaeological Museum in Poznań). No additional permits were required for this research, which complied with all relevant regulations.

**Fig 5:**
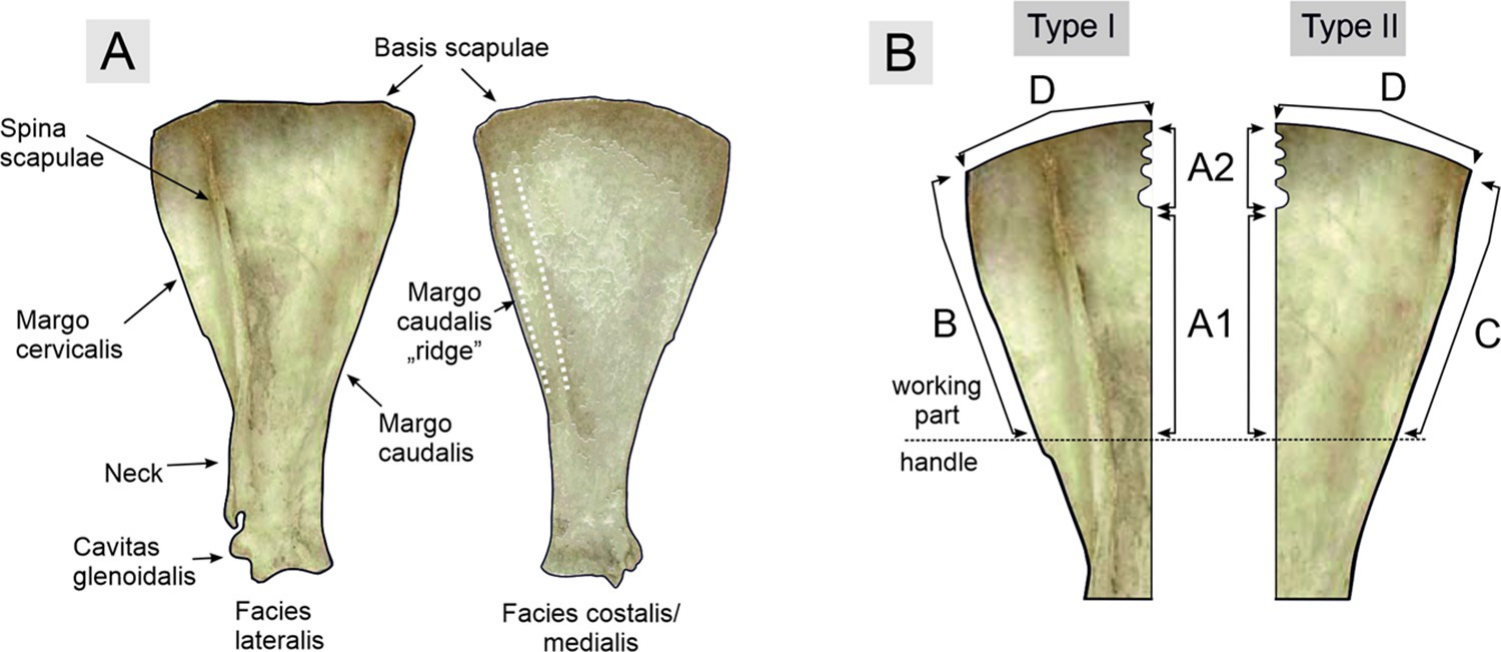
Osteological terminology used in the article (A) and working edges numbering scheme of Bruszczewo-type tools of both distinguished morphological types (B).

The applied technological terminology is based on osteological nomenclature (cf. Fig 5A) and relevant prior studies [e.g., 56–61]. The working edges identified on the analysed tools are marked with numerals (Fig 5B). In the classification system applied to notched-bone scapulae, Roman numerals were used to mark their morphological types, whereas Arabic numerals were employed to introduce classification based on functional types.

**Fig 6:**
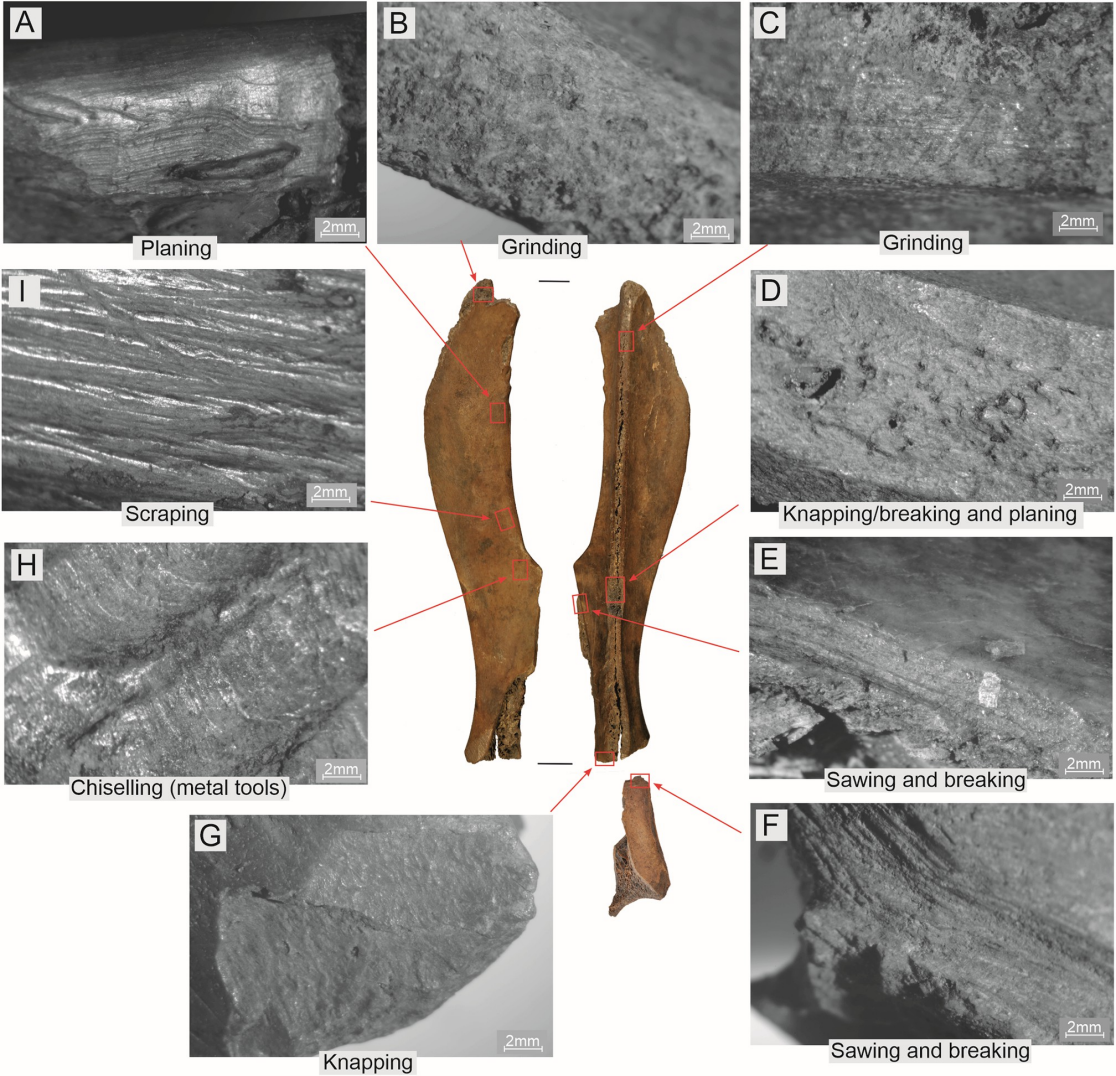
Examples of technological traces observed on Bruszczewo-type tools.

The traceological analyses were performed using two microscopes. Studies of the artefacts’ state of preservation and initial analysis of the technological and use-wear traces were performed using a Nikon SMZ-745T microscope (up to 65× magnification) fitted with a Delta Pix Invenio 6EIII camera. The latter was used to obtain the photomicrographs presented in Fig 6. Observations of polish were conducted using a Zeiss Axioscope 5 Vario microscope equipped with an Axiocam 208 camera. The photomicrographs presented in Figs 10–15 and 18–20 were also produced using this equipment.

The criteria for use-wear identification and terminology applied in the traceological (functional) studies were based on the published conceptual system [e.g., 2, 56, 62–75]—adjusted to comply with the analysis’ purposes and requirements.

The classification of use-wear traces visible on the Bruszczewo-type tools presented in this paper was devised based on direct observations made during microscopic studies and a detailed analysis of over 700 microphotographs captured during the research.

The scanning electron microscope (SEM) analysis was conducted using an SEM/FIB Quanta 3D FEG equipped with a Schottky FEG thermal field emitter module with an LVSED detector and accelerating voltages of 200V up to 30 kV. The microscope was operated at a spatial resolution of 1.2 nm in three optional modes (high vacuum, low vacuum, and ESEM). The SEM analyses involved fragments of the four artefacts’ working edges. Owing to the size of the microscope’s vacuum chamber, these samples were small—about 1 cm long and about 0.6 cm wide (this also applies to the samples subjected to the scanning electron microscopy and energy dispersive X-rays spectroscopy [SEM-EDX] analysis). Notably, SEM analysis was used predominantly to investigate changes in the topography and texture of bone surfaces and discover residues, as its utility for analysing polish is highly limited (i.e., [76–78]).

The presence of inorganic residue on five of the artefacts was verified through SEM-EDX analysis using a scanning electron microscope (SEM; LEO Electron Microscopy Ltd, England, model no. 1430 VP from 2001). The device is intended for analysing the surface topography of solids and enables controlled vacuum conditions (1e270 Pa), allowing low-conductivity and low-hydration samples to be investigated without prior treatment (e.g. applying a conductive coating). The device was connected to a Quantax 200 energy-dispersive X-ray spectrometer (EDX) with an XFlash 4010 detector (Bruker AXS, Germany in 2008), through which the elemental composition of various parts of the objects could be determined.

The search for organic residues on Bruszczewo-type tools using optical microscopes was implemented in two stages. Stage one was conducted at the BIAX Consult Biological Archaeology and Landscape Reconstruction Laboratory in the Netherlands. Two implements preserved in small fragments (nos. 21 and 25, cf. S1 Table), which had previously been traceologically analysed, were examined. Both artefacts were first viewed under a Leica binocular incident light microscope at magnifications from 6× to 50×. Subsequently, selected fibres preserved within the bone matrix of both artefacts, were subjected to further identification. They were fixed onto glass slides using a permanent medium. Analyses were performed using transmitted light microscopy with polarised light at a maximum magnification of 400×.

Stage two was conducted at the DANTE- Diet and Ancient Technology Laboratory at the Sapienza University of Rome. The analysis was performed on four tools (nos. 8, 25, 33, and 41, cf. S1 Table). The last two specimens were neither washed nor subjected to microscopic analysis to maximally decrease the likelihood of impurities and destruction of potential residues. The studies were conducted using a ZEISS Axio-Zoom stereo-microscope (10× to 165×) and ZEISS Axio Scope A1 (100× to 400×) for low- and high-magnification observation, respectively. The first of these devices was used to create the microphotographs presented in Figs 9A–9F; 17A. The other microscope was used to create the microphotographs presented in Fig 17B and 17C. The microphotographs of residues presented in Fig 17D–17H were made using the Zeiss Axio Imager microscope. The study of use-wear traces and residues was performed in situ to provide complementary data to understand the modalities of how the tools were used.

The morphoqualitative features of the residues (e.g. colour, appearance, inclusions, consistency, and birefringence) were interpreted by directly comparing them with the experimental residues from the collection at the DANTE laboratory. These include varied residues used for hafting and/or binding (e.g. natural and ochre-dyed strings of hide and sinews; plant fibres; and adhesive compounds such as bee wax, resin, bitumen, and animal glue). Only residues exhibiting features such as patination, smearing, flattening, and directionality were considered reliable for functional interpretation.

Further, artefacts were sampled for residue observation under transmitted light. Each bone tool was dry and wet brushed with distilled water, each time using a new toothbrush to remove all residues trapped in the tool surfaces’ pits and crevices. Starch and phytolith extraction procedures were consistent with the combined protocol described by Pagan-Jimenez [79]. The extracted residues were mounted on glass slides using a 50:50 solution of water and glycerol and observed under transmitted light microscopy using a ZEISS IMAGER with a magnification of up to 630×.

Taphonomical observations were made according to relevant literature concerning artefacts made of bone and antler [80–82]. Importantly, these include potential changes that might have resulted from the influence of the sediment wherein the artefacts were deposited [83], trampling by animals and humans [84], and other potential alterations caused by plants and animals [85–87].

#### Experimental research programme

The studies on the function of Bruszczewo-type tools required the planning and implementation of a series of archaeological experiments. The programme of these experiments was structured considering all likely hypotheses regarding their utilisation (including those discussed in the literature) to be tested using a base of experimental products found at the Institute of Archaeology, Nicolaus Copernicus Univ. in Toruń. Currently, this collection includes approximately 200 osseous items which allowed us to omit several activities that had already been tested using similar tool forms (e.g. various techniques and stages of hide working). The developed experimental research programme covered activities not performed before that were considered to be likely predicated on (1) the observations made based on the preliminary characteristics of the use-wear traces preserved on the artefacts, and (2) the test results obtained for organic and inorganic residues identified on their surfaces.

To conduct the experimental studies, 24 replicas of Bruszczewo-type tools were crafted using tools and techniques analogous to those applied to the artefacts. Twenty of the products were made of porcine scapulae, two were made of bovine scapulae, and two were made of cervine scapulae. Eleven of the best-crafted specimens (nine made of porcine scapulae and products made of cow and red deer bones) were used in the following activities: (1) flax fibre extraction; (2) pressing the wool thread when making selvedge; (3) wool felting; (4) colouring the wooden surface with chalk- and ochre-based mineral pigments; (5) daubing a wattle; (6) pinching the coils, as well as smoothing/slipping of a ceramic surface during pot-making; (7) separating seeds from ears of wheat (threshing); (8) separating peas from pods (threshing); and (9) brain tanning.

*1*. *Flax fibre extraction*: Bundles of cut, dried, and crushed flax were placed in a container with water and soaked for two weeks. Subsequently, the plants were dried, and the experiment was initiated. It involved breaking the flax stems as well as breaking apart the fibres by pulling them between the blade of the tool and the thumb. The experiment involved two replicas of Bruszczewo-type knives that were efficiently employed for 60 min and 90 min.
*2*. *Pressing the wool thread when making selvedge*: This experiment aimed to test the possible use of Bruszczewo-type knives as weaving tools. Sheep wool yarn (100%) was used as the warp and weft. The work was completed in 30 min.
*3*. *Wool-felting*: This experiment involved the use of wool from *wrzosówka* sheep (a Polish breed of domestic sheep), which was felted on a mould using hot water to obtain the shape of a vessel (a bowl). The osseous implement was employed to press the raw material, while water was squeezed out simultaneously, and the surface was felted. The experiment was completed in 90 minutes.
*4*. *Colouring with mineral chalk- and ochre-based pigments*: This experiment aimed to test the possibility of using Bruszczewo-type tools for plastering wood (a wall made of pine slats) with mineral pigments, namely, ochre and chalk. The ‘paint’ was prepared by mixing the pigments with melted animal fat. In the experiment involving chalk paint (whitening), one tool was worked with for 90 minutes. Covering the wall with the ‘ochre’ paint involved two bone products, one of which was worked with for 60 minutes, and the other one for 90 minutes. One side of the tools’ working edges was used as a contact surface, and the performed motion of use resembled smoothing conducted perpendicularly to the working edge’s line.
*5*. *Pugging a wattle*: In the experiment, replicas of the analysed tools were used for distributing and smoothing out daub (a mixture of clay and sand) on a wattle wall. The experiment involved two products that were worked with for 60 minutes.
*6*. *Pottery making*: The experimental tool was employed for pinching the coils, as well as for smoothing out/slipping the surface of a ceramic pot when it was being shaped. The exact time of working with the tool is unknown as it was used ‘as necessary’. However, it was no shorter than approximately an hour.
*7*. *Threshing cereals*: In this experiment, two replicas of Bruszczewo-type tools were used to thresh recently harvested bread wheat. The work was performed at the time of harvest (early August) and involved drawing the working edge of a given tool repeatedly along a cereal bundle (approximately 6 cm in diameter) laid on a wooden plate (a debarked tree trunk approximately 30 cm in diameter). The tool was applied repeatedly until the ears of the bread wheat separated into free grains and chaff, which usually meant that it had to be drawn out about four–six times (depending on the thickness of the processed bundle). Both specimens employed in the experiment were used for approximately 3 1/2 hours.
*8*. *Threshing legumes (peas)*: The process of threshing peas was similar to that of threshing cereals. The experiment was conducted in July using plants from organic farming (the field was weedy). During the experiment, a replica of a Bruszczewo-type tool was used. The tool’s working edge was repeatedly drawn along a plant bundle placed on a wooden plate. Whole plants were used, and no pods were separated from the stems. The tool was applied repeatedly until the peas had separated from the pods, which—as in the case of wheat—usually meant that it had to be drawn out approximately four–six times. The experiment was conducted for an hour, and the work with the tool was considered highly effective (certainly more effective than the process of separating peas from the pods by hand).
*9*. *Brain-tanning*: In this experiment, a tool made of a bovine scapula was used to rub porcine brain matter into a recently cleaned cowhide (with the flesh and connective tissue remains scraped off) for approximately an hour.

## Results

### Results of zooarchaeological studies

For detailed results of the zooarchaeological analysis, cf. Makowiecki 2015 [88] and S1 Table.

Forty of the Bruszczewo-type tools were made of scapulae, while one of them was made of an equine mandible. Regarding the implements made of scapulae, the bone species used could be determined in the case of 24 specimens. As many as 14 of them were crafted from bovine scapulae; in 8 cases, the right scapula was used (hereafter referred to as R), whereas in 6 cases, the left scapula was used (hereafter referred to as L). Two other specimens were most likely (but not definitely) also made from bovine scapulae (2 × R). Three tools (only assumed in one case) were crafted using the bones of a red deer (2 × R and 1 × L). Additionally, one more tool was identified as the right scapula of a bovine or red deer. Two specimens were made of bones from wild boars (2 × R), and one was made from either wild boar or domestic pig (R). One tool was made of an equine scapula (whether it was the right or left one could not be determined). For the remaining 16 implements made of scapulae (4 × R, 4 × L, 8 × indefinite), the species of the raw material were not identified.

### Taphonomic condition of artefacts

The state of preservation of the analysed specimens was generally extremely good and allowed for reliable traceological analyses. However, some were preserved in fragments, and several others had various types of post-depositional modifications, such as surface discolouration, iron-rich precipitates, and exfoliation of the outer surface of the bone. Erosion caused by plant roots was observed only in some cases. Further, modern (post-discovery) destruction was visible on the surface of some artefacts, usually small cracks, scars, and striations—attributable to transport or improper maintenance.

### Results of the technological studies—biographies of tools

Microscopic analysis allowed us to identify numerous types of technological traces using the microphotographs presented in Fig 6. The (S1 Table) provides a detailed profile of this type of damage identified on the surfaces of specific artefacts.

Owing to the abundance of the collection and the fact that it involves forms representing different stages of the production process and utilisation of the products in question, reconstructing the chain of operations applied in the crafting process and interpreting the rules applied to utilisation, including the description of the repairs carried out on the working edges, was possible to a high degree of accuracy.

**Fig 7:**
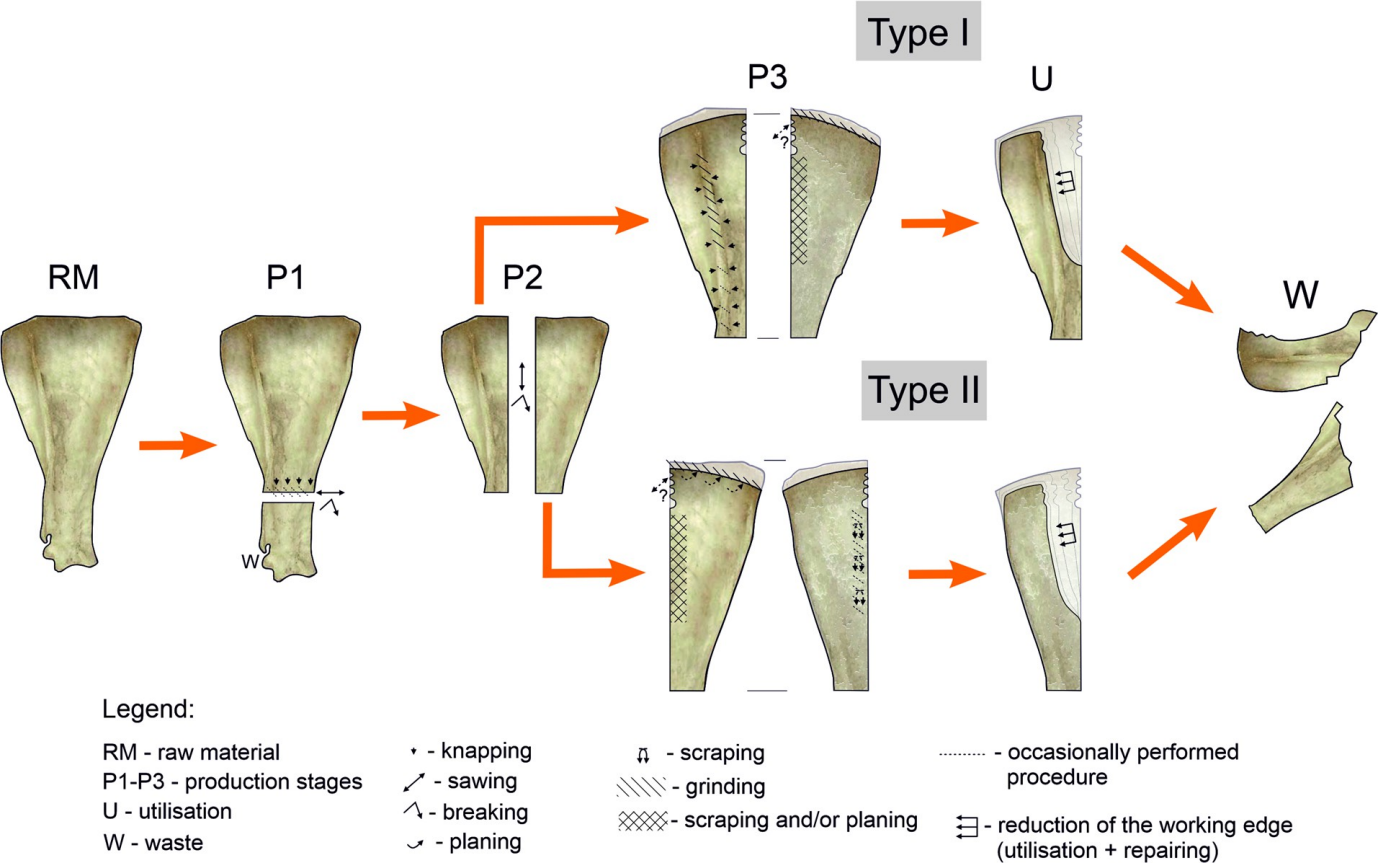
Reconstructed ‘biography’ of Bruszczewo-type tools.

The first stage in the chain of activities performed during the manufacture of the Bruszczewo-type tools (aside from the stage wherein the raw material was cleaned) was the removal of most of the neck of the processed scapula, including the glenoid fossa (*Cavitas glenoidalis*) below the nutrient foramen (stages P1 –Fig 7). This was performed by making an omnidirectional incision in the bone and breaking off the unwanted piece (Fig 6F). The waste created in this process was unearthed as single finds in layers at the site in Bruszczewo (Fig 4: 2–4). The resultant irregularities on the edge (fracturing) were usually removed by knapping (Fig 6G) and breaking (and in exceptional cases by grinding).

The subsequent stage in the chain of operations while crafting the products (P2 –Fig 7) involved dividing the worked scapula into two parts, more or less along the axis (the line running from the *Cavitas glenoidalis* to the *Basis scapulae*—between the *Spina scapulae* and *Margo caudalis*). This was performed by uni- or bilaterally presawing the raw material down to the level of the bone’s spongy portion, followed by breaking it along the incision line (Fig 6E). In rare cases, the neck with the *Cavitas glenoidalis* was removed during this stage (stage P1), as evidenced by a single find of this type of waste with visible remains of the longitudinal cutting of the raw material typical of stage P2 (Fig 4: 3). In the collection, only one artefact shows the described production stage, though in this case the preliminary processing also involved the *Basis scapulae* (Fig 3:6).

Stage P3 in the production of the tools covers the activities performed to provide their surfaces with the final shape and form the working edges. The course of this stage differed for forms made of scapula parts that involved the *Margo cervicalis* and *Margo caudalis*, allowing us to distinguish two variants of Bruszczewo-type tools (Fig 7). Specimens made of the parts containing the *Margo cervicalis* were classified as type I. In the collection, 12 tools of this type could be distinguished, 9 of which were made of the right scapula. Type II specimens were crafted from the parts containing the *Margo caudalis*. Overall, 11 tools of this type have been identified, 8 of which were made of the right bone.

Regarding type I tools, the *Spina scapulae* was worked predominantly on the *Facies lateralis*, broken off/knapped and/or whittled across the entire length (Fig 6D), and then also frequently ground (Fig 6C). The latter activity was conducted particularly meticulously at the working edge (cf. Figs 6B; 7), with a fragment of the *Spina scapulae* located on the handles not being ground.

The working edges A1 and A2 of the type I tools usually bear no processing traces on the *Facies lateralis*. However, on the *Facies costalis*, they were formed in all cases. In this respect, significant differences were observed in the techniques applied between tools made of the left and right scapulae. All the specimens made of left scapulae had their A1 edges ground on the *Facies costalis*. The tools made of the right scapulae were ‘sharpened’ using flat surface scraping and/or planing (Fig 6A and 6I), and only occasionally by grinding (1 case). More differences were observed in the case of rules applied during the forming of working edge D. On the tools made of the right scapulae, they were ground at a high angle on the *Facies costalis* (Fig 6B) to create a blade on the *Facies lateralis*, whereas the opposite approach was used in the case of the specimens made of the left scapulae.

For the type II tools, the processing performed in stage P3 was limited to creating working edges. However, this was performed in a manner somewhat different from that applied to type I products (Fig 7). In contrast to the latter, in this case, the preparation of edge A1 basically involved ‘abrading’ the surface of the *Facies lateralis* exclusively, though just as in the case of type I specimens in tools made of right scapulae, predominantly planing and/or scraping was used (with one different case during which grinding was also applied), whereas for those made of left scapulae, surface grinding was applied. Working edges D in the products of the discussed type were prepared just as in the case of type I specimens made of left scapulae, that is, by grinding at a high angle on the *Facies lateralis* to form a working edge on the *Facies costalis*.

Working edges A2 have been preserved only in a few cases (on four specimens of each type); how they were made remains unclear (primarily owing to the well-developed use-wear traces that cover them). However, in some cases, they were most likely formed using the sawing technique.

The processing of working edges B and C was only occasional and was most likely determined by the need to make minor corrections to these edges or by the necessity to clean the raw material. This was performed using highly diverse techniques, such as scraping, planing, grinding, or chiselling. Similar rules were applied to finish the neck parts in both types of implements, which formed their handles. The features of some types of technological traces observed on the Bruszczewo-type tools (e.g. Fig 6H; in this case, the shape, depth and repeatability of the chiselling negatives) indicate that they were manufactured using metal tools. Nevertheless, the scope of application of forms made from this raw material remains unspecified.

Regarding the final outcome, the work undertaken as part of stage P3 in the production of the objects in question yielded complete forms of implements, examples of which (non-used) were identified in the analysed collection (type I–Fig 3: 1, 4; type II–Fig 3: 2). This stage concludes the manufacturing stage in the biography of Bruszczewo-type tools and opens the utilisation stage which—in some cases—started long before all the working edges had been finished. This is because on some of the products, traces of use were observed on edges A, B, or C before the final shaping of edge D (type I–Fig 3: 1, 3; type II–Fig 3: 5).

The utilisation stage of the Bruszczewo-type tools (stage U–Fig 7) involves a slow reduction of their working edges as a result of progressive use-wear damage and repairs. This comprised reprocessing the working edges using various techniques such as scraping, planing, chiselling, and grinding. Occasionally, the old working edge A1 was cut off, as in the case of the specimen presented in Fig 4: 1. The utilisation of a given tool was likely concluded by the time edge A had been completely damaged, which for type I tools, meant that the level of the *Spina scapulae* had been reached (Fig 2: 1, 2; 4: 5–7), while in the case of type II tools, this indicated that the *Margo caudalis* ‘ridge’ on the *Facies costalis* had been reached (Fig 2: 4). The tools were also abandoned (stage W–Fig 7) owing to mechanical damage of various types, such as the working part being broken off (Fig 4: 5–10).

### Functional analyses results

**Fig 8:**
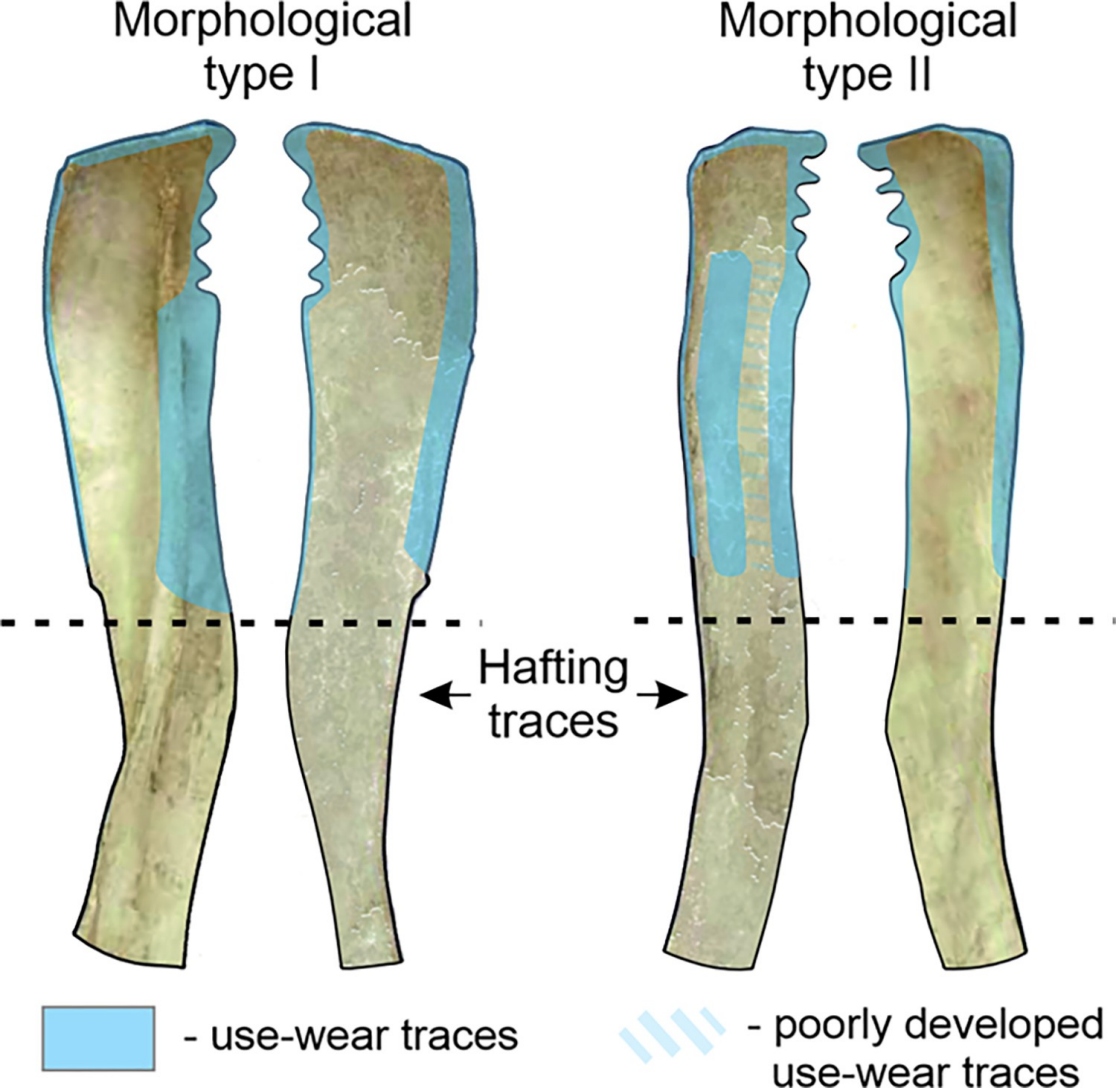
Bruszczewo-type tools: Use-wear traces’ location.

The first stage in the functional analyses that the Bruszczewo-type tools were subjected to was intended to examine the use-wear changes readable on their surfaces using small magnifications and optical microscopes. Consequently, the scope of occurrence of use-wear traces was determined for specific working edges (Fig 8).

**Fig 9:**
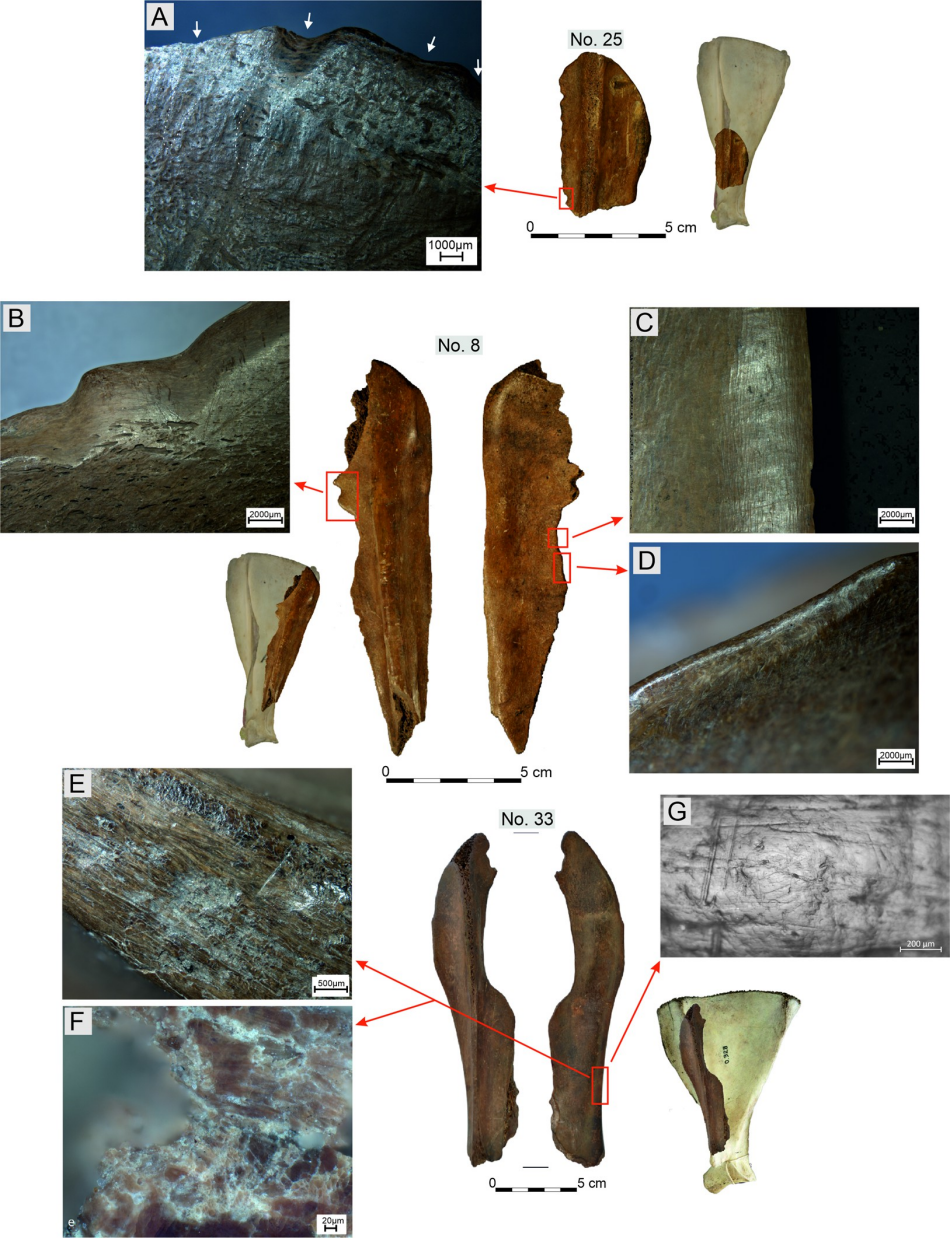
Bruszczewo-type tools: Examples of the macro-wear traces.

This stage of research allowed us to identify several types of macro-traces typical of the products discussed. Apart from the rounding of the working edge encountered on tools of several functional types (e.g. Fig 9A and 9D), other less common use-related modifications occurred:

1. Denticulate deformations of the tool’s cutting edge: U-shaped depressions occurring in series on the cutting edges were formed as the bone fibres were compressed because of being pressed by a medium-hard material, which did not cut the surface. These deformities lend the cutting edge a micro-serrated course (usually only locally; Fig 9A–compression marked with arrows).
2. Linear deformations of the tool’s working edge (linear depressions): shallow linear depressions with a trough-like cross-section visible on the working edge (its lateral surfaces). Their lengths and widths vary. Typically, the starting point for the individual linear depressions was the cutting edge (Fig 9A–marked with a dashed line). Traces of this type co-occur and coincide with linear traces and compressed residues. Their origin is analogous to that of the denticulate deformations of the tool’s cutting edge.
3. Flattening of the surface on the working edge, not affecting natural depressions: continuous flattening of the working edge formed as a result of bone fibre compression on a larger portion of the working edge (denticulated–Fig 9B or not denticulated–Fig 9C and 9D). Surfaces of this kind seem to be ribbed (at small magnifications–Fig 9C), while the working edge alone is faceted (Fig 9D).
4. Rounding outside the working edges: nonlinear rounding of the microrelief of the bone outside the working edge area (readable on the necks of the scapulae, i.e. the probable handles of the specimens–Fig 9G). These were accompanied by some hair-like multidirectional linear traces and more legible deep grooves perpendicular to the tool axis. The anatomical structures of the bone (osteons) are quite visible. In the described areas, traces of compression of the bone fibres (nonlinear depressions/crush outs and cracks–Fig 9E and 9F) and discolouration (darkening) of the surface were also observed.

The described use-wear traces exhibit varying intensities on most of the better-preserved Bruszczewo-type tools.

#### Classification of use-wear traces (micro-traces) observed on tools

**Fig 10:**
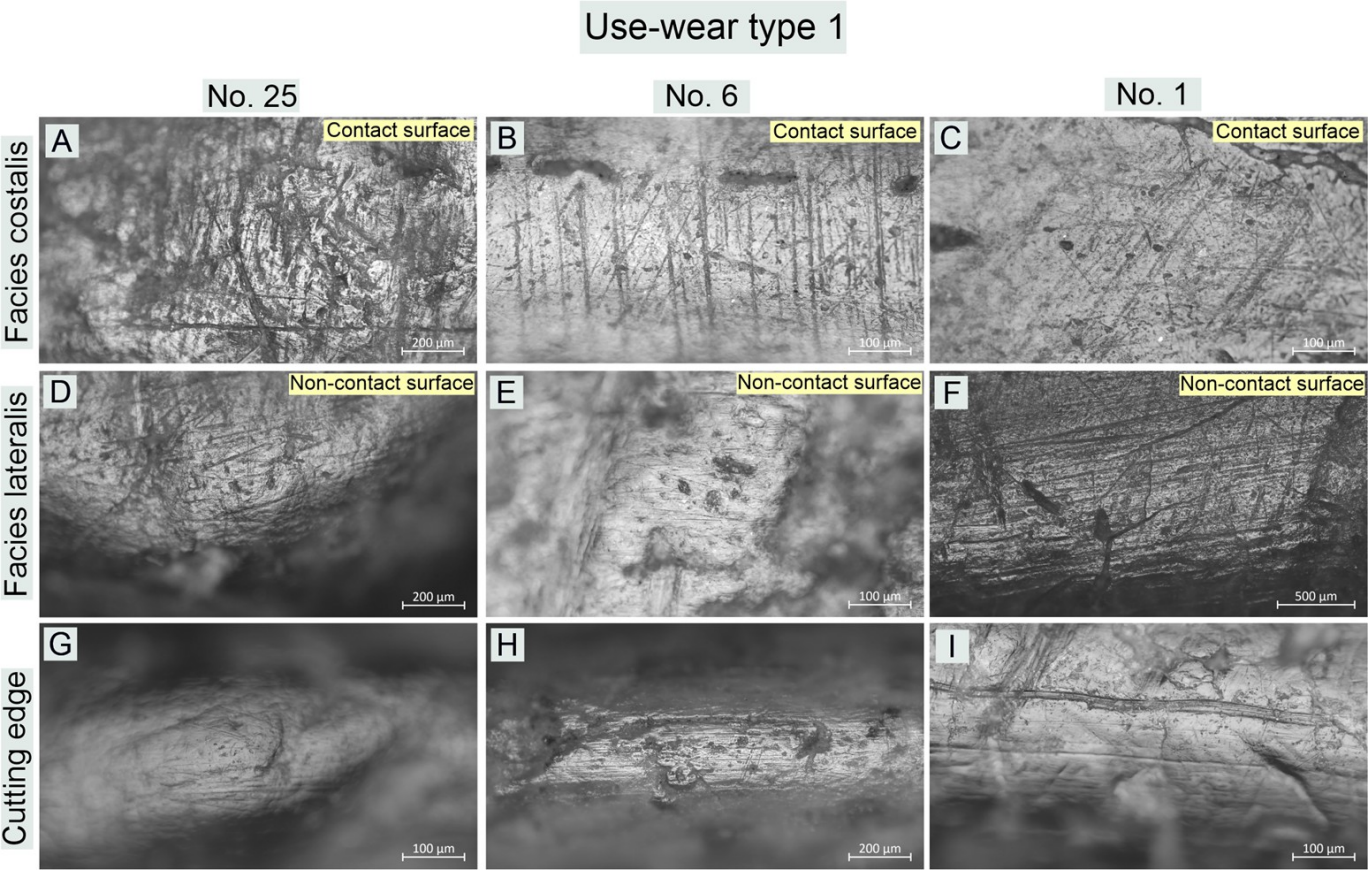
Bruszczewo-type tools, working edge A1: Examples of use-wear type 1 traces.

**Fig 11:**
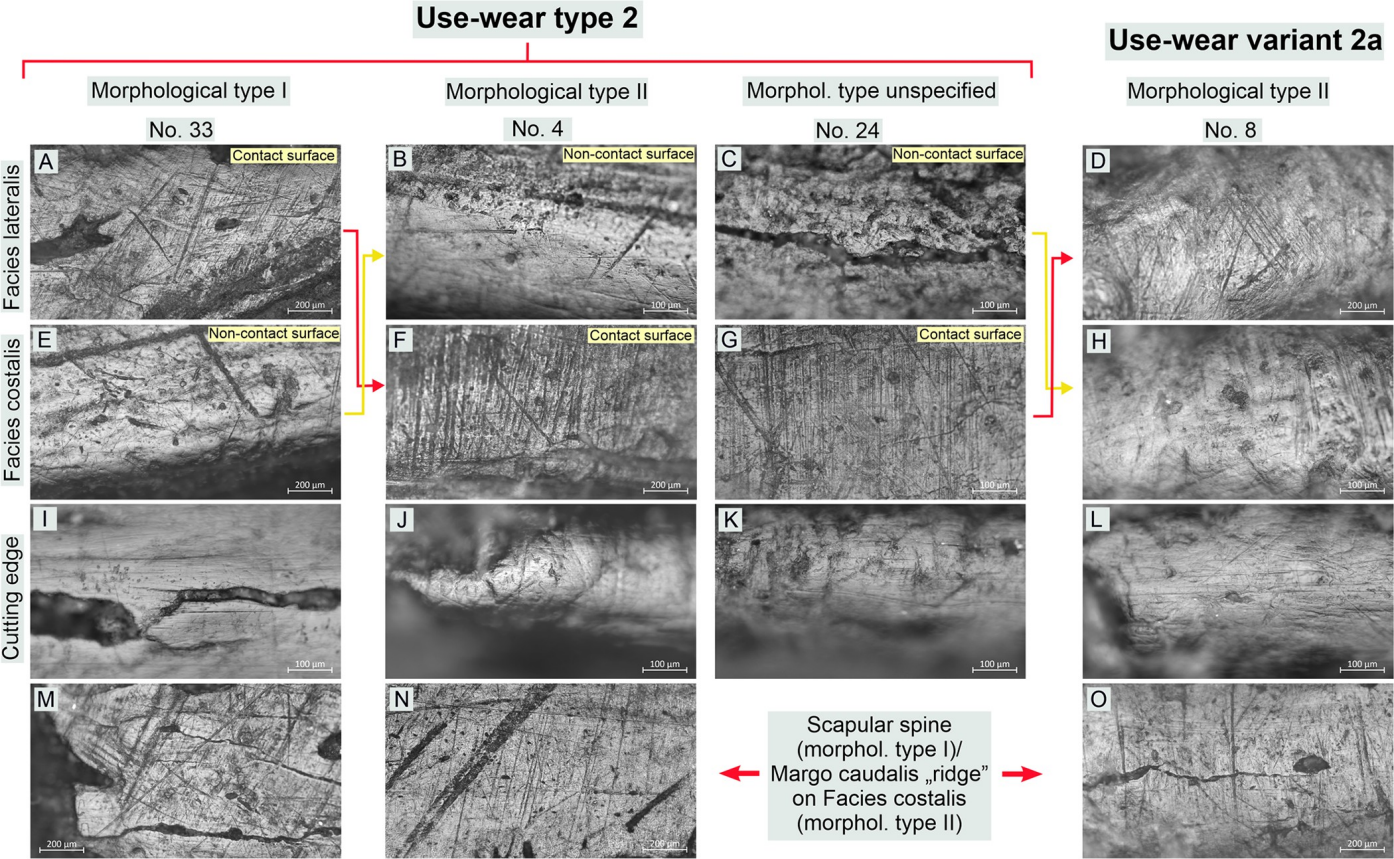
Bruszczewo-type tools, working edge A1: Examples of use-wear type 2 and variant 2a traces.

**Fig 12:**
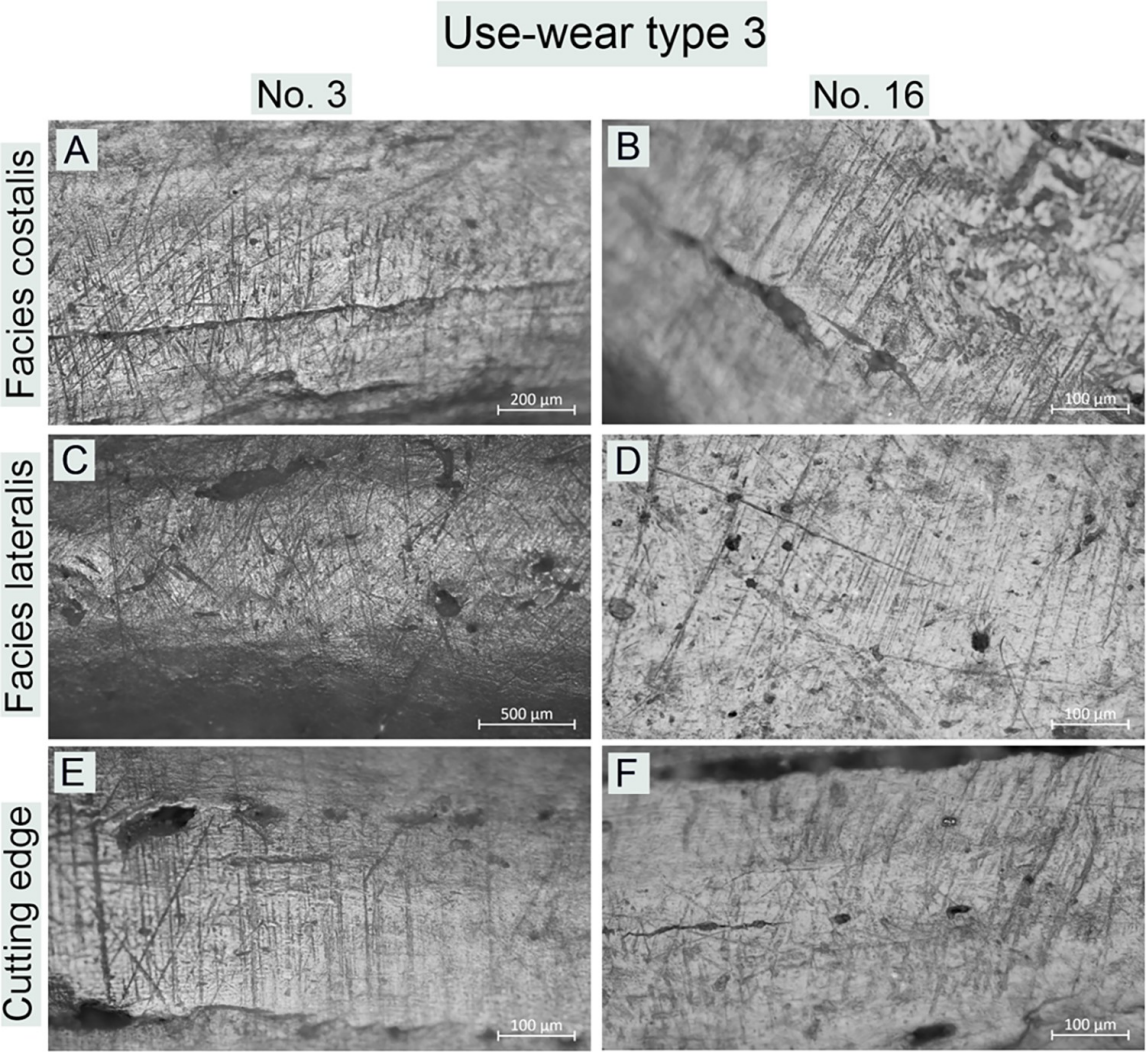
Bruszczewo-type tools, working edge A1: Examples of use-wear type 3 traces.

**Fig 13:**
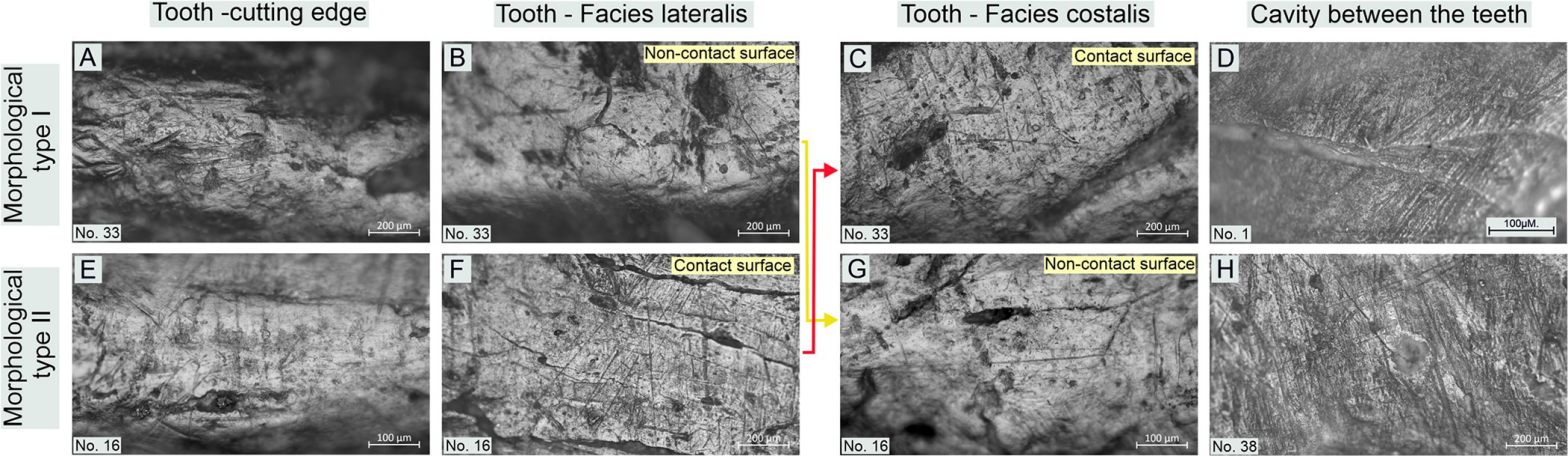
Bruszczewo-type tools, working edge A2: Examples of use-wear traces.

**Fig 14:**
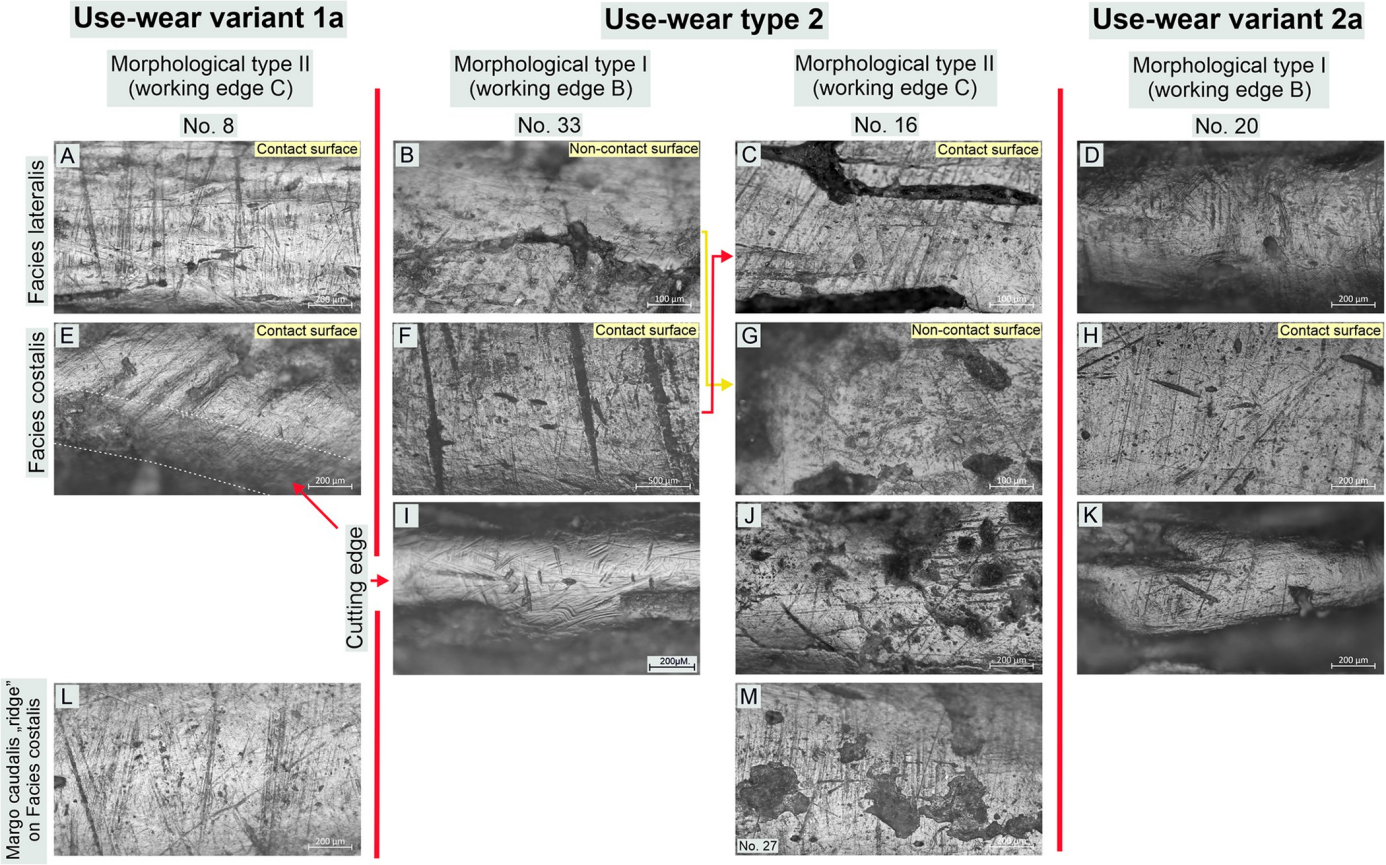
Bruszczewo-type tools, working edges B and C: Examples of use-wear type 2 and variants 1a and 2a traces.

**Fig 15:**
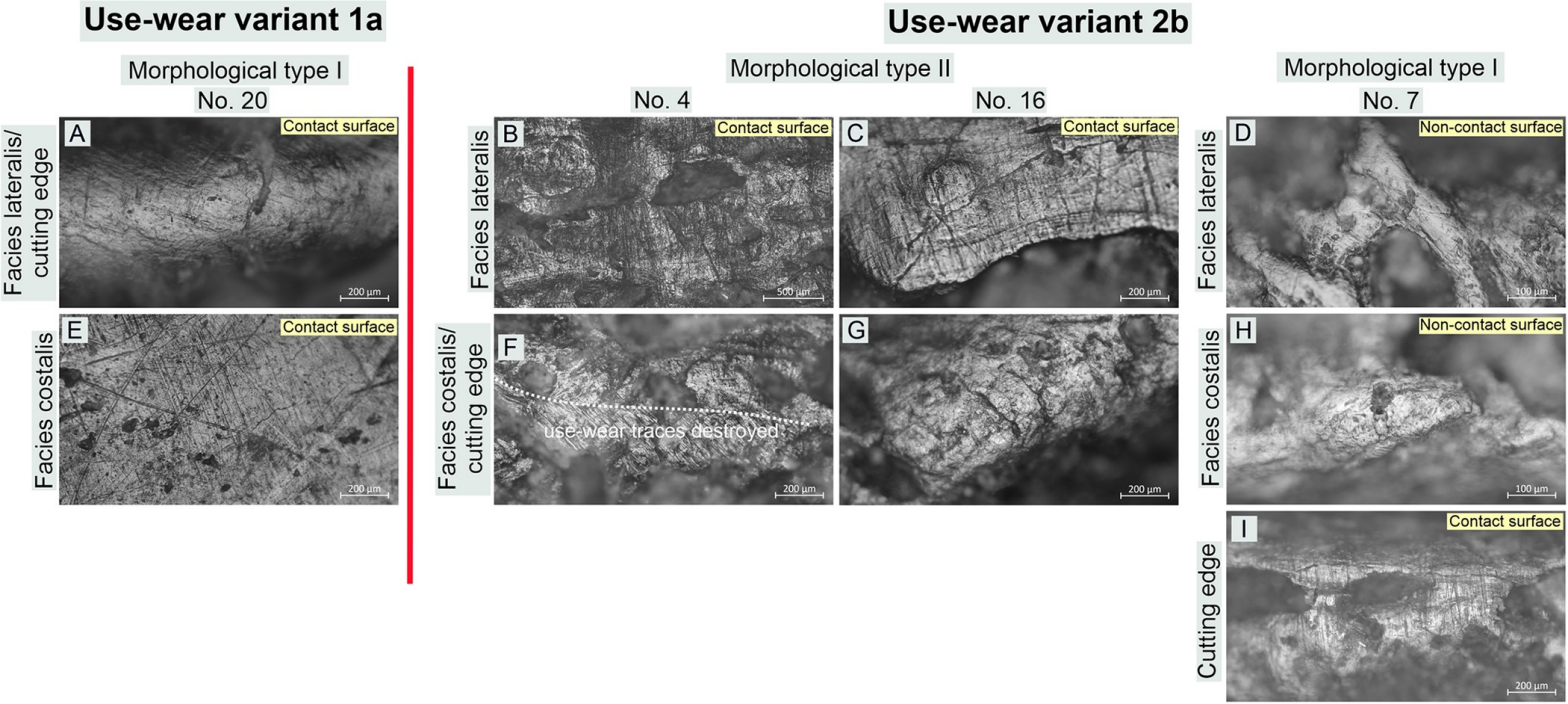
Bruszczewo-type tools, working edge D: Examples of use-wear variants 1a and 2b traces.

The traceological studies to which the artefacts were subjected exhibited relatively high uniformity in the use-wear macro-traces readable on the working edges. Nevertheless, the studies of the micro-traces (polish and linear traces) present on these implements using metallographic microscopes at high magnifications allowed us to observe some inconsistencies between specific tools. They are readable even at the same working edge level (cf. Figs 10–15).

These differences serve as the foundation for classifying the use-wear traces visible on Bruszczewo-type tools into three main types and three subtypes:

*Use-wear type 1*. On the contact faces of the working edges classified as this functional type, abrasive polish with a highly invasive degree of intrusion was observed, predominantly in the upper parts of the microrelief and penetrating the entire microrelief, only on the specimens that were most worked with (Fig 10A–10C). Its topography is heterogeneous. The high points are highly polished and basically flat. The areas located deep inside the microrelief are covered by polish to a significantly smaller degree or show none at all. This causes the topography to be slightly pitted. The microrelief of the polish is regular, and its texture in the uppermost areas is basically smooth. The area deep inside the microrelief remains slightly rough. The polish is accompanied by linear traces oriented perpendicularly or oblique to the line of the working edge. These are predominantly black or slightly filled in striations or grooves with highly ‘ragged’ yet rounded edges and uneven bottoms. They are usually about 400–700 μm long and about 5–10 μm wide. They occur in isolation or in groups and intersect.

The characteristics of the use-wear traces observed on non-contact faces of the working edges differ. Here, the microrelief in the polished areas is significantly more diverse (albeit still regular), while the clear rounding and ‘melting’ of its individual elements (including the highest points) renders the profile of the polish ‘domed’ (Fig 10D and 10E). The microtopography of the polish is more homogeneous than on the contact faces and lacks the typical abrasiveness (flatting out/obliteration of the uppermost points). This was observed in only one case (no. 1 –Fig 10F). The texture of the polish can vary, though it is particularly rough on the above-mentioned tool (no. 1).

The most characteristic and distinctive feature of the non-contact faces of the working edges of this functional type is the presence of linear traces oriented parallel to the cutting edge’s line (Fig 10D–10F). Usually, these are short, black, and filled striations, rarely longer than 200 μm and usually about 2–3 μm wide, which are an integral part of the linearity of the polish (Fig 10D and 10E). Significantly more abrasive linear traces were observed only in the case of tool no. 1, including invasive grooves up to several centimetres long and even up to 80 μm wide (Fig 10F).

Similarly, the cutting edges of the products of the discussed functional type were covered by a polish oriented in line with the blade of the tools (Fig 10G–10I). Its profile is similar to that described for non-contact faces. It has a homogeneous domed and corrugated topography, and its texture is smooth. The polish is related to identically oriented linear traces (whose features are analogous to those visible on noncontact faces).

*Variant 1a*. Only one tool was identified as bearing use-wear damage of this kind (no. 8, cf. S1 Table). The polish and linear traces observed on the contact side of its working edge are analogous to those described above, in the case of functional type 1 (Fig 14A). The situation is similar for the cutting edge and the non-contact face of the working edge, though the wear-use traces observed are oblique and not parallel to the line of the tool’s cutting edge, as in the case of the type 1 specimens (Fig 14E).

*Use-wear type 2*. The use-wear traces observed on the contact surfaces of this type of implement are analogous (or highly similar) to those visible on the contact faces of the specimens classified as type 1. Usually, their key element is a bright polish with flat—sometimes slightly corrugated—heterogeneous topography, and slightly rough texture (Figs 11A, 11F, 11G; 14C and 14F). It is related to perpendicular linear traces in the form of (primarily) black hair-like striations of uneven edges, up to about 1000–1200 μm long (usually about 700 μm) and about 4–8 μm wide (Fig 11G). There were also single occurrences of tools on which such striations were more abrasive (their width ranging from 10 to 20 μm–Figs 11F and 14C) or more subtle (about 500 μm long and about 3μm wide–Fig 11A).

Traits that were most distinctive for traces typical of products classified as this functional type were identified on their non-contact faces (Figs 11B, 11C, 11E; 14B and 14G). The surfaces of the tools are clearly rounded in that area and covered with a polish that usually penetrates the relief of the raw material throughout. Its topography is domed and corrugated (yet nearly flat in some areas) and homogeneous, whereas its texture is very smooth. The microrelief of the polished surfaces is regular, with the highest points visibly slightly domed. The most characteristic feature is that linear traces are either absent or represented solely by single multidirectional striations of varying characteristics (most likely mostly of post-depositional origin).

On the cutting edges of the tools classified as this functional type traces analogous to those described for functional type 1 were recorded, that is, a polish of a domed topography, a smooth texture, and linear traces (black and filled striations) oriented in line with the working edge (Figs 11I–11K; 14I and 14J).

*Variants 2a and 2b*. The differences in the characteristics of the use-wear traces observed on the implements of type 2 and subtype 2a are readable on the non-contact faces of the working edge. The polish visible on the variant 2a products (unlike the one present on the tools of type 2) is clearly linear (perpendicular) and coincides with subtle (usually not numerous) linear traces (hair-like and black striations, and single ‘larger’ grooves–Figs 11H; 14D).

Variant 2b tools differ from type 2 products solely in that they lack the linear traces on the cutting edge oriented in line with the working edge (Fig 15).

*Use-wear type 3*. On both faces of the working edges of the products classified as this functional type identical use-wear traces were observed, whose characteristics are analogous to those described for the contact surfaces of the implements of use-wear types 1 and 2 (Fig 12A–12D). Therefore, the noncontact surfaces of the working edges cannot be distinguished. The cutting edges exhibit no linear traces oriented in the line of the working edges. Here, only striations occurred, oriented perpendicularly, whose profile was comparable to that of traces of this type identified on both faces of the working edges (Fig 12E and 12F).

#### Use-wear profiles of the working edges

*Working edge A1*. The working edges A1 of 19 tools were observed to bear use-wear traces the characteristics of which allowed us to classify them (cf. S1 Table). Six of them exhibit damage typical of functional type 1 (Fig 10). These are three specimens of morphological type I, two of which (nos. 1 and 25) have well developed use-wear traces, whereas one of them (no. 30) is at an early stage of utilisation and is therefore functionally ambiguous. The situation was similar for the sole tool of morphological type II covered here, on which poorly developed use-wear traces were observed (no. 28). The collection is complemented with two products of unspecified morphological type (fragments of tools) on which clearly readable use-wear traces were recorded (nos. 6 and 15). On one of the knives of morphological type I (no. 25) included in this functional group, in the area where the *Spina scapulae* were originally located, use-wear damage was observed with traits similar to those described for contact surfaces. This indicates that this area was probably used analogously.

On the working edges A1 of six implements we observed traces corresponding to type 2 use-wear, and in four other cases, they corresponded to variant 2a (Fig 11; cf. S1 Table). In the group of tools with use-wear type 2, we included three tools of morphological type I (nos. 33, 40, and 20). Specimen no. 40 is severely damaged, whereas tool no. 20 was repaired and then used only for a brief period; therefore, the observed use-wear traces did not exhibit all the distinctive characteristics. Moreover, two implements of morphological type II (nos. 4 and 21) and one unspecified form (no. 24) were classified into this category.

Functional variant 2a comprises three tools of morphological type II (nos. 8, 38, and 27) and one of morphological type I (no. 23). Specimens nos. 8 and 38 are residual forms, while tool no. 23 is severely damaged, and product no. 27 was used for a very short time. These factors may have had a key impact on the characteristics of the use-wear traces visible on the products classified in this group.

The arrangement of the contact edges in the depicted tool group changes depending on the classification of a given morphological type. In the case of the first one (I), the role of the contact surfaces was played by the *Facies lateralis* of the tools. Here, not only do the distinctive use-wear traces cover the area of edge A1, but also the area of the *Spina scapulae*, rendering the degree of intrusion of the polish highly invasive (even up to several centimetres–Fig 8). As for the implements classified as morphological type II, the role of contact surfaces was played by the *Facies costalis*, with traces related to the use of this edge usually affecting the *Margo caudalis* ‘ridge’ as well (Fig 14L, 14M; cf. Fig 8).

The described arrangement of contact surfaces has also been preserved on most of the products classified as functional subtype 2a. Only in the case of tool no. 8 was the role of the contact surface fulfilled by the *Facies lateralis*.

Working edges A1 of three implements (nos. 16, 3, and 31) bear traces that correspond to use-wear type 3 (Fig 12; cf. S1 Table). The last specimen is severely damaged and its classification is uncertain. All the products represent morphological type II.

*Working edge A2*. The analysed collection involves seven tools with working edges A2 whose state of preservation allowed us to conduct a microscopic analysis. This group comprises three specimens each of morphological types I (nos. 1, 33, and 40) and II (nos. 8, 16, and 38), as well as one product unspecified in this respect (no. 14, cf. S1 Table).

The use-wear traces identified on two implements (nos. 8 and 40) did not allow to classification in terms of function. In the other cases, the observed damage corresponds (in the general profile) to type 2 use-wear (Fig 13). However, noteworthily, on some working edges A2 (or fragments thereof), the characteristics of the damage visible on both sides of the teeth were quite similar. On their contact faces, a few linear traces are also present on the opposite surfaces, which are considered non-contact. These characteristics of the traces make them closer to variant 2a. The situation is similar for the tips of some teeth, on which striations oriented in the line of the tool blade are poorly readable, which can be considered a trait of variant 2b (Fig 13E).

The use-wear traces observed in the recesses between the teeth are different from those observed on their tips. Here, an invasive polish of flat topography and a heterogeneous, rough texture occurred, coinciding with numerous linear traces in the form of perpendicular black and (occasionally) filled striations (Fig 13D and 13H). Regarding characteristics, this damage corresponds to that described for the severely utilised contact surfaces of tools of all the distinctive functional types.

*Working edges B and C*. In the analysed collection, there were 18 implements with working edges B/C preserved well enough to subject them to traceological analysis (cf. S1 Table). In this assemblage, eight specimens of morphological type I were identified (nos. 1, 9, 20, 23, 25, 33, 37, and 40) along with the same number of tools of morphological type II (nos. 4, 8, 16, 21, 27, 31, 34, and 38) and two products whose state of preservation rendered classification as any of these types impossible (nos. 24 and 26).

Based on the conducted microscopic studies, one of the specimens (no. 8) was found to bear use-wear damage typical of use-wear variant 1a (Fig 14A and 14E). On nine tools traces corresponding to use-wear type 2 are found (cf. Fig 14), six of which are products of morphological type I (nos. 1, 9, 23, 25, 33, and 40), and three others represent morphological type II (nos. 16, 21, and 27). Additionally, on one specimen (no. 20), traces typical of use-wear variant 2a were identified (Fig 14D, 14H and 14K). The other seven products remained unspecified in terms of their functional type for various reasons (severe damage and low degree of use-wear damage).

As in the case of working edge A1, the arrangement of the contact surfaces of working edges B/C of the analysed tools varies depending on the morphological type to which they are assigned. In the case of specimens representing type I, the role of the contact face of working edge B was played by the *Facies costalis*, whereas in tools of morphological type II (working edge C), it was played by the *Facies lateralis* (cf. Fig 14).

*Working edge D*. The traceological analysis of the working edges marked as D was hindered by the fact that they had been finished/repaired by grinding to reveal the spongy bone, thereby exposing a structure far more susceptible to the influence of post-depositional factors. Therefore, these edges are usually more damaged than the other parts of the analysed implements.

The working edges D of 11 products were identified as bearing use-wear traces (cf. S1 Table). However, only six of them, were well-preserved enough that it was possible to examine them in detail and classify them. Three of the tools in this group (all of morphological type II; nos. 4, 16, and 27) show damage typical of the use-wear variant 2b (cf. Fig 15). One can also possibly include one more tool in this functional category (no. 7, morphological type I). This specimen has a peculiar working edge that was ground at a right angle to its *Facies costalis* and *Facies lateralis*. This renders the use-wear traces of traits typical of contact sides, which are readable for functional type 2b, on the *Facies lateralis*, and are visible on the blade and overlap with the *Facies lateralis* only to a small degree (cf. Fig 15D, 14H and 15I). In the collection, one tool was additionally identified with traces typical of use-wear variant 1a (no. 20, cf. Fig 15A and 15E) and one on which damage typical of variant 2a occurred (no. 21). However, considering the severe obliteration of the blade on this specimen, this cannot be confirmed.

#### SEM

**Fig 16:**
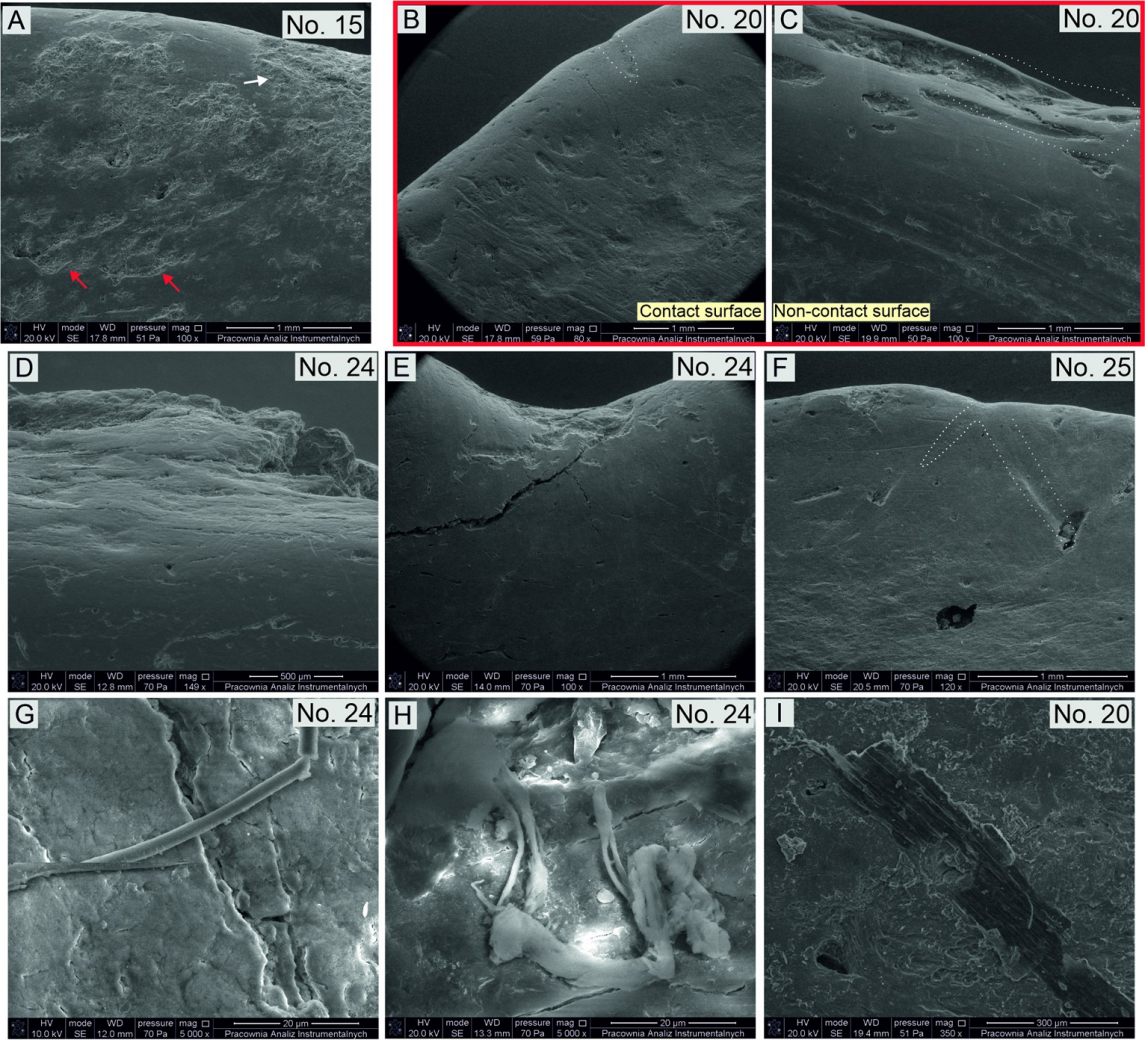
Examples of use-wear damage and residues identified based on SEM analysis.

First, the SEM analysis confirmed the presence of significant post-depositional changes on the working edges of the analysed artefacts (Fig 16A–red arrows) and the possibility of identifying residues of various kinds (Fig 16A–white arrows).

Moreover, SEM proved to be a useful tool for examining changes in the texture and topography of the working edge surfaces. In this respect, the presence of clear differences was positively verified between the cutting edges (flat topography, texture completely smooth, no perpendicular linear traces), the contact surfaces of the working edge (perpendicular striations present, texture clearly rougher, topography highly diverse–Fig 16B), and the non-contact surfaces of the working edge (microrelief of the raw material preserved better, texture only slightly rough, common linear traces oriented parallel–Fig 16C).

Using SEM on the working edges of the Bruszczewo-type tools numerous use-wear traces caused by ‘compression’ were also recorded. Areas where the fibres of the raw material were distinctively compressed were noted on the cutting edges of the artefacts. They are readable in the form of crush-outs and multiple depressions in the surface emphasised by numerous cracks (Fig 16C–marked with a dotted line; 16D). The compression areas are also visible between the teeth of edge A2, where they coincide with subtle perpendicular linear streaks (Fig 16E). Moreover, on the working edges of the examined artefacts, the presence of numerous linear depressions caused by compression was confirmed (Fig 16B and 16F–marked with a dotted line).

One of the fundamental objectives of using SEM to investigate the Bruszczewo-type tools was to study the residues. Unfortunately, because of the small size of the examined samples, the analyses were not successful (in places selected for analysis they were not preserved). On the investigated fragments of working edges, organic and inorganic remains of various kinds were observed (Fig 16G–16I), which were all deemed to be contaminations not related to the function of the implements.

### Results of residue studies

#### SEM-EDX

In all five cases, SEM-EDX studies of fragments of the working edges of the Bruszczewo-type tools yielded similar results. Table 1 presents an exemplary distribution of elements in one of the specimens.

**Table 1:** Example results of SEM-EDX analysis of the Bruszczewo-type tool working edge (no. 25).

Mass percent (%)															
Spectrum	C	O	Na	Mg	Al	Si	P	S	Cl	K	Ca	Ti	Mn	Fe	Ba
38063	4.23	46.17	1.77	1.50	7.61	20.57	3.08	0.34	0.27	1.77	7.30	0.33	1.11	3.95	-
38064	1.14	57.77	1.33	0.73	1.16	1.51	9.64	0.13	-	0.34	24.26	-	0.73	1.26	-
38065	1.28	53.01	1.24	0.81	3.78	8.79	6.79	0.11	0.12	0.75	16.86	0.18	3.12	3.15	-
38067	1.89	54.97	1.13	0.51	0.50	0.49	11.37	0.15	-	-	27.29	-	0.32	1.38	-
38068	2.61	51.11	0.43	-	1.06	0.44	10.03	1.45	-	-	27.35	-	0.37	1.26	3.90
38069	2.39	52.75	0.76	-	1.08	0.39	11.83	0.61	-	-	28.72	-	0.28	1.20	-
38070	4.36	54.41	0.46	0.28	2.31	3.01	7.43	2.41	-	0.34	21.37	-	0.28	3.35	-
38071	2.51	52.34	0.46	0.18	1.12	0.61	11.14	1.66	-	-	26.23	-	0.19	1.56	2.02

In addition to phosphorus, calcium, and oxygen coming from hydroxyapatite (the elements which make up the bones), point inclusions were observed, composed of compounds of aluminium and silicon, as well as metals such as sodium, magnesium, potassium, iron, and titanium, and rarely encountered manganese and barium. To investigate the provenance of these additions, a comparative analysis of earth and pottery samples from the site was conducted.

The analysis of the soil from the site (cf. Table 2) showed high content of silicon (3.29%–46.35% w/w) and aluminium (0.80%–4.80% w/w) from silicates and aluminosilicates, as well as the presence of metals typical of silt in the form of oxides: iron oxide (0.69%–2.09% w/s), sodium oxide (up to 0.67% w/w), potassium oxide (0.10–1.24% w/w), magnesium oxide (0.05–0.64% w/w), and titanium oxide (0–54.93%), as well as a small amount of sulphur (< 0.65% w/w). This elemental composition is common in the silt of the Greater Polish Lowland. At one point, manganese was also observed (1.59% w/w); though traces of barium were not observed in these samples.

**Table 2:** Example results of SEM-EDX analysis of the soil from the site in Bruszczewo.

Mass percent (%)														
Spectrum	C	O	Na	Mg	Al	Si	P	S	Cl	K	Ca	Ti	Mn	Fe
38270	1.99	46.91	0.37	0.64	4.80	40.19	0.09	0.30	-	0.93	1.43	0.26	-	2.09
38271	1.14	47.99	0.67	0.51	2.43	45.04	0.15	0.17	-	0.42	0.51	-	-	0.97
38272	1.05	49.25	0.24	0.21	1.72	45.65	-	0.21	-	0.33	0.39	0.17	-	0.76
38274	11.24	68.90	-	0.28	2.48	7.51	0.03	0.65	0.06	1.24	5.60	0.19	-	1.83
38276	0.00	8.79	-	0.09	3.33	29.09	-	-	-	0.65	1.33	54.93	-	1.80
38277	0.14	42.47	-	0.05	0.80	3.29	-	-	-	0.10	0.22	22.99	1.59	28.35
38278	0.34	50.07	-	0.16	1.69	46.35	-	0.13	-	0.26	0.32	-	-	0.69

Regarding the analysis of pottery fragments from Bruszczewo (Table 3), apart from the standard components of clay and silt, a high barium content of up to 3.78% w/w was recorded, which, in several cases, was higher than the calcium, sodium, potassium, magnesium, or titanium contents.

**Table 3:** Example results of SEM-EDX analysis of the pottery from Bruszczewo.

Mass percent (%)												
Spectrum	C	O	Mg	Al	Si	P	S	K	Ca	Ti	Fe	Ba
40501	0.92	65.01	0.36	7.25	22.27	0.08	0.29	1.47	0.59	0.56	1.19	-
40502	9.84	58.98	0.23	1.90	28.19	0.03	0.14	0.20	0.11	0.09	0.26	-
40503	0.45	59.37	0.44	7.98	21.50	0.05	3.05	1.43	1.05	-	1.68	3.00
40504	0.76	58.16	0.73	8.88	27.24	0.27	0.51	1.39	0.59	-	1.10	0.37
40508	2.50	49.51	0.34	7.51	23.72	0.90	1.98	3.27	3.65	0.94	4.69	0.99
40509	3.10	45.87	0.99	9.71	28.79	1.16	0.58	3.29	2.06	1.62	2.82	-
40510	2.38	47.76	0.31	6.52	21.25	1.60	1.74	2.63	2.86	-	9.16	3.78

#### Optical microscopy

In the first stage of this research, which was conducted at the BIAX Laboratory, numerous fungal thread-like structures (referred to as fungal mycelia or hyphae) were observed embedded within the spongy tissue of the bone. In both analysed artefacts, tiny fragments of plant fibres (from a few to several in each artefact) and a couple of cotton and synthetic fibres were also found. One of the plant fibres was dyed blue. The fibres were also embedded in the pockets of spongy bone tissue, often trapped in the fungal hyphae network.

Microscopic examination of the selected fibres in polarised light revealed the presence of morphological and anatomical features characteristic of plant bast fibres such as x-cross markings, dislocations, and fine lumina (central canals). The fibres were c. 10 μm in diameter and were preserved as often c. 3 mm-long fragments. The anatomical features of the plant fibres observed under the polarised light microscope are typical of various species, such as flax, hemp, and nettle. As no evidence of microfibrillar orientation was observed, the species determination of the Bruszczewo material is uncertain. The diameter of the fibres and fine lumina suggest that they possibly originated from flax.

**Fig 17:**
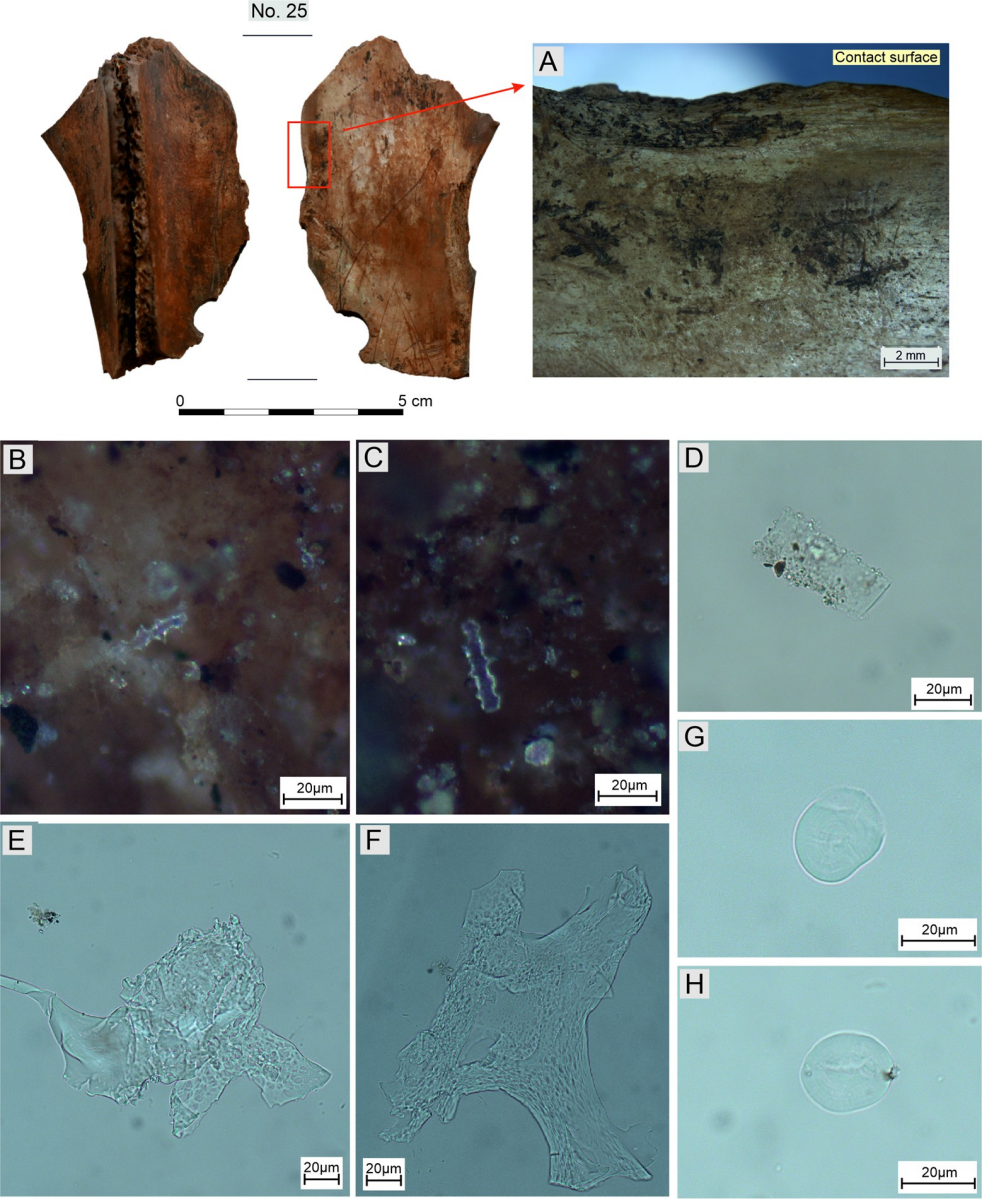
Examples of organic residues observed on Bruszczewo-type tools.

In the course of the research conducted at the DANTE Laboratory, macro- and/or micro-residues were identified on the surface of all five analysed tools. They were visible in the working edge areas of the tools, marked by visible use modifications (Fig 17A). In the case of the first tool (no. 41), two elongated dendritic phytoliths were observed *in situ* at high magnification, still adhering to the surfaces and located relatively far from the denticulation (Fig 17B and 17C). These phytoliths are of particular interest because their morphology has been experimentally linked to the processing of cereal inflorescences [89]. Four other elongated phytoliths, common in cereal stems and leaves, were identified under transmitted light (Fig 17D). Additionally, eight starch granules were identified on the tool and associated with legumes of the *Fabaceae* family (Fig 17G) and cereals of the *Triticeae* tribe (Fig 17H), based on grain morphology and distribution. Two fragments of spongy parenchyma tissues have also been identified (Fig 17E and 17F). This type of tissue is common in seeds and was found in abundance after the experimental processing of legumes.

In addition to tool no. 41, starch granules and phytoliths were identified on only one other tool (no. 25). The other tools had only starch or no residues at all.

### Results of the experimental studies

**Fig 18:**
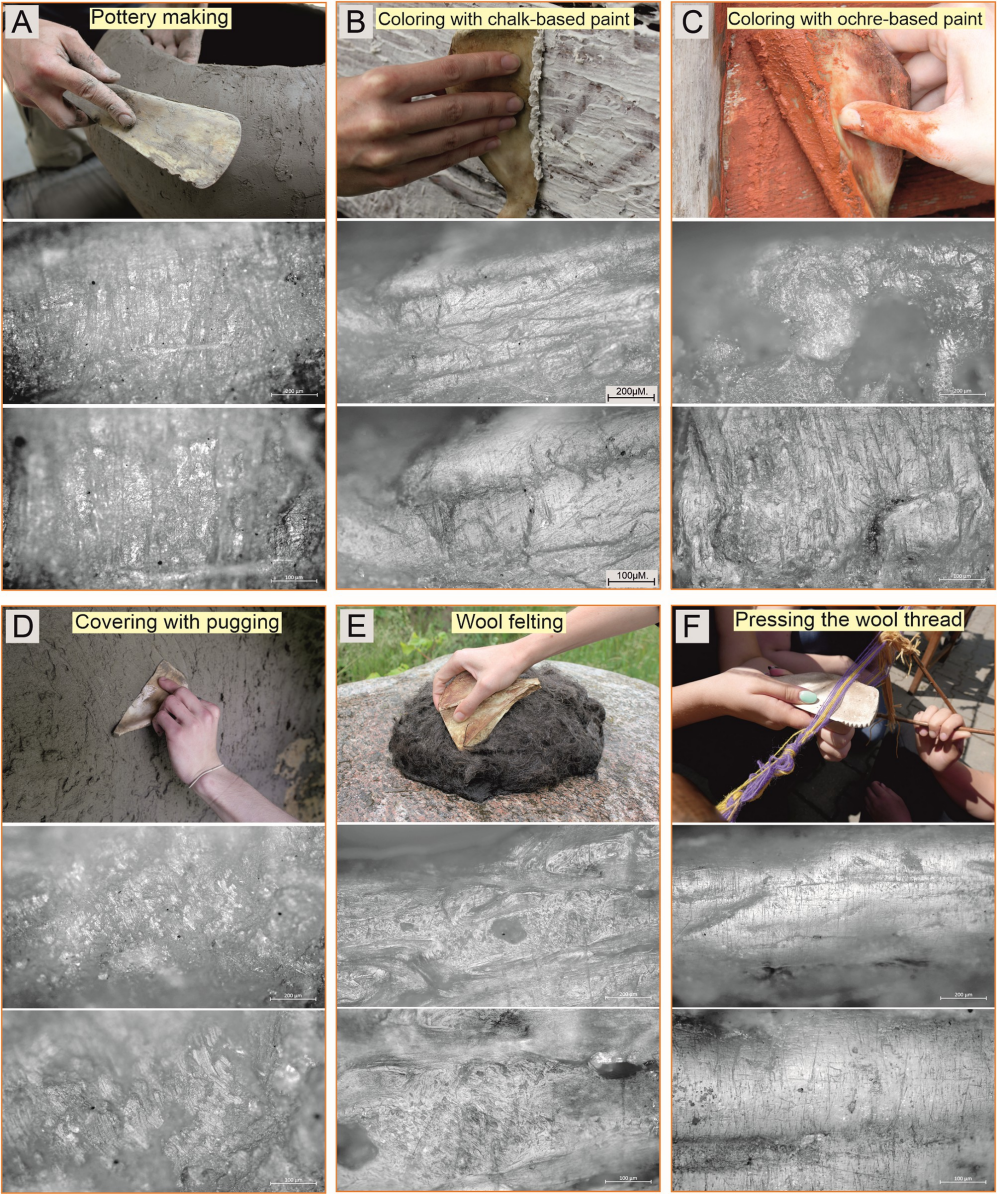
Examples of the use-wear traces observed on the working edges of the experimental tools: Pottery making, colouring with chalk-based and ochre-based paints, covering with pugging, wool felting, and pressing the wool thread.

**Fig 19:**
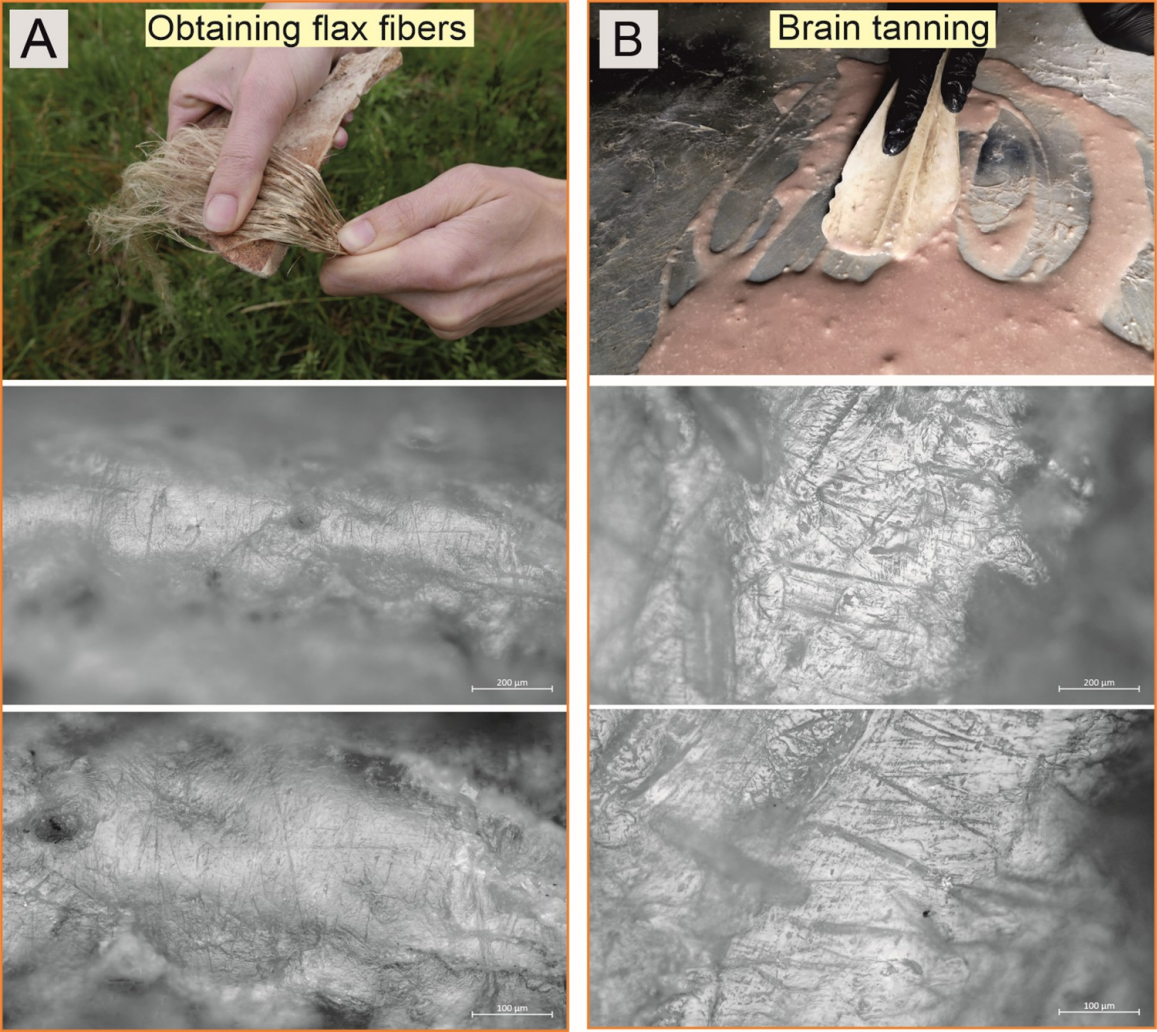
Examples of the use-wear traces observed on the working edges of the experimental tools: Obtaining flax fibres, and brain tanning.

**Fig 20:**
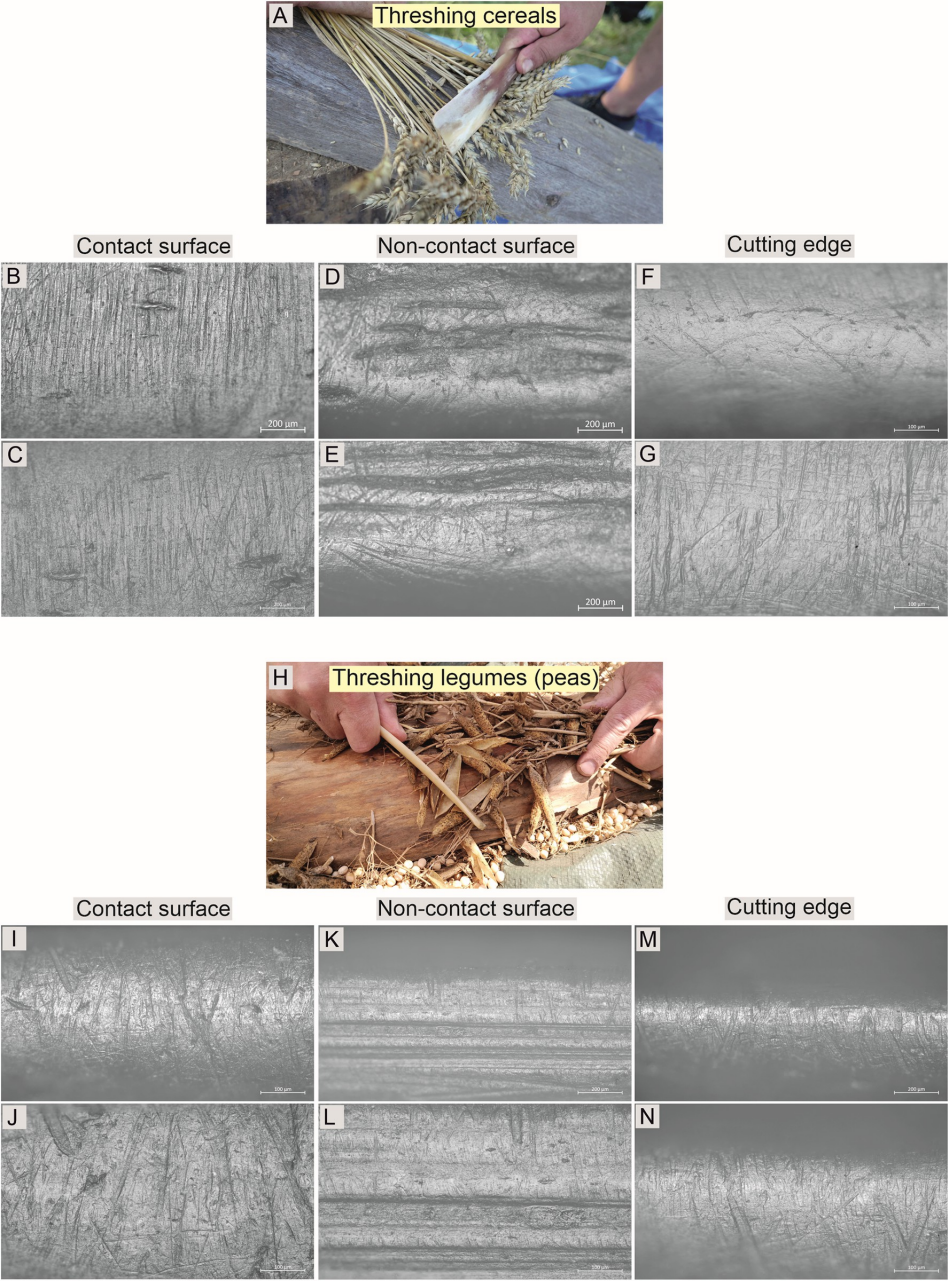
Examples of the use-wear traces observed on the working edges of the experimental tools: Threshing cereals and legumes (peas).

The studies of the reference collection available at the IA NCU in Toruń showed no presence of experimental tools bearing use-wear traces analogous to those observed on the Bruszczewo specimens. Based on the traceological analysis of the products used during the experimental programme devised for the reported studies, the use-wear traces formed on the specimens employed for specific activities were deemed highly diverse (cf. Figs 18–20). Table 4 lists their general characteristics. Below, only the damage observed on the implements used for threshing cereals and legumes (peas; Fig 20) is presented in further detail, as this is important for the reported studies.

**Table 4:** Characteristics of the use-wear traces observed on experimental tools: Selected examples.

Experiment	Use-wear traces	Figure
Making pottery	Linear roughening of the working edge, associated with wide and deep linear grooves oriented perpendicularly. Spread, pale polish with heterogeneous micro-topography and irregular micro-relief.	Fig 18A
Colouring with chalk-based paint	Quite bright, linear polish with domed, homogeneous micro-topography, quite regular microrelief and rounded highest points. Its texture is slightly rough. It is connected with delicate, sparse hairline linear traces oriented perpendicularly (predominantly filled in striations).	Fig 18B
Colouring with ochre-based paint	Quite bright linear polish covering the upper parts of the microrelief, slightly rough texture, domed, heterogeneous topography. Its microrelief is quite regular, the highest points slightly rounded. Characteristics are deep and wide, irregular linear grooves, and black striations.	Fig 18C
Covering with pugging	The polish is built of linear bands, destroying the microrelief.	Fig 18D
Wool felting	Basic feature–clearly emphasizing the structure of osteons and covering them with a bright, non-linear polish with a smooth texture, domed/heterogeneous topography and irregular microrelief.	Fig 18E
Pressing the wool thread	Bright polish with a basically flat, homogeneous topography, regular microrelief, and slightly rough texture; associated with perpendicular linear traces (short and thin black striations with uneven edges).	Fig 18F
Obtaining flax fibres	Linear matting with a rough texture, strongly rounding the micro-relief. Heterogeneous microtopography, irregular microrelief. Characteristics are numerous, usually very short, shallow hairline linear traces oriented perpendicularly (mainly black striations). The degree of intrusion of polish is marginal, almost only cutting edge.	Fig 19A
Brain tanning	Very bright polish with flat topography, smooth texture associated with not too many linear traces (black grooves and striations) with various characteristics.	Fig 19B

On the contact faces of the tools employed in the experimental cereal threshing (Fig 20A), a bright polish with a heterogeneous (locally flat) topography, regular microrelief, and basically flat high points was observed. Its texture is slightly rough, and it has an invasive degree of intrusion (Fig 20B and 20C). This polish is associated with numerous linear traces oriented perpendicularly to the line of the working edge. These are predominantly black striations and grooves, with uneven edges and bottoms, usually about 400 micrometres (up to about 700 micrometres) long.

On the non-contact faces of the working edges, the observed (quite bright) polish completely covers the microrelief of the raw material (except for the deepest parts—Fig 20D and 20E). Its topography is essentially flat, slightly domed, and homogeneous, and the texture is slightly rough. The microrelief is basically illegible. This polish coincides with rare multidirectional linear traces (irregular, short grooves). In several places, no linear traces are observed.

On the cutting edges of the tools, a polish with a flat/slightly domed and homogeneous topography was observed. Its texture is almost smooth, and it obliterates the microrelief of the raw material. No linear traces appear here, except for some poorly legible (shallow), filled-in, multidirectional grooves (Fig 20F). In this part of the working edge of one of the tools used to work in two directions (both side surfaces of the working edge are contact surfaces), traces analogous to those visible on the contact surface can be observed on the cutting edge (Fig 20G). Traces parallel to them were also identified on the cutting edges in some areas (Fig 20G).

The traces observed on the tool used for experimental pea threshing (Fig 20H) are, to some extent similar to those described for cereal threshing however, significant differences exist. On the contact side of the working edge of the tool used for pea threshing, a bright polish with a heterogeneous topography (more domed than in the case of cereals), regular microrelief, and rounded high points were observed. Its texture is slightly rough, and it has an invasive degree of intrusion (Fig 20I and 20J). This polish is associated with numerous linear traces oriented perpendicular and oblique to the working edge’s line. Their arrangement is decidedly more ‘chaotic’ than in the case of traces typical for cereal threshing, and they intersect much more often. These are primarily black striations and grooves with uneven edges and bottoms, significantly shorter than in the case of traces after threshing cereals, with a length usually reaching about 100 μm to a maximum of 200 μm. However, this may be attributable to the shape of the working edge of the tool used for processing peas, that is, its greater rounding. However, the important aspect in the discussed case is that significantly more irregular grooves were observed with a width of up to 20 micrometres, which (next to the chaotic arrangement) seem to be the most characteristic element of linear traces resulting from threshing peas.

On the noncontact face of the working edge of the described tool, the observed (quite bright) polish completely covers the microrelief of the raw material only on the highest locations of the microrelief (Fig 20K and 20L). Its topography is slightly domed and homogeneous, and its texture is slightly rough (in places completely smooth). This polish coincides with numerous, very short linear traces: black striations oriented perpendicularly to the line of the working edge. Their length is only about 30 micrometres, with a width not exceeding 1 micrometre. These traces are legible only in the immediate vicinity of the cutting edge and disappear at deeper depths.

The traces on the cutting edge of the experimental tool used for threshing peas are highly similar to those observed on the contact side of its working edge (Fig 20M and 20N) and to those locally visible on specimens used for threshing cereals (cf. Fig 20G).

## Discussion

Characterising the discussed group of scapular tools from European sites, Northe [9] distinguished two basic types, namely—specimens whose main working edge is not notched (type A) and those whose main working edge is notched and that show perforations for repairing the haft (type B). In line with this classification, the vast majority of Bruszczewo artefacts should be classified as type A (e.g. Figs 2: 3; 3: 2) or a type intermediate between A and B, that is, implements whose main working edge on the vast part of its length bears no notches (edge A1), whereas they occur only on a small part of it (edge A2; e.g. Fig 2: 1, 2, 4). In the analysed collection of osseous products, there was only one tool with a fully recessed working edge that could be classified as type B (Fig 4: 10). However, this specimen has no drilled perforations for mounting onto the haft. Hence, the products from Bruszczewo evade the Northe classification system, and seemingly, the categorisation into two morphological types based on technological criteria introduced in this article is far more appropriate.

Our technological studies are the first detailed research of this kind involving Bruszczewo-type tools. Previous observations of this profile, which referred to the manner whereby the scapula notched implements were manufactured, are limited to Northe’s publication [9], wherein they assume the form of general suggestions and speculations not supported by direct traceological/functional studies of archaeological artefacts. Therefore, the results of the technological studies presented here cannot be compared with the findings of similar analyses of different collections. Nevertheless, the studies reported herein clearly show that the Bruszczewo-type tools were made in accordance with a chain of operations that was developed in detail and strictly followed, which we were successful in fully reconstructing owing to the large number of analysed tools, semi-products, and production waste. Noteworthily, metal tools were undoubtedly applied in some of the activities performed in the process of creating the discussed implements.

The implements in question were manufactured primarily using bovine scapulae. Determining whether we are addressing an actual preference for this animal taxon or if the observed regularity is attributable to the availability of these bones is challenging. Apart from this type of bone, scapulae of other animal species were also used, including those of wild animals such as the red deer. A similar situation has been observed for other European sites where scapula-notched implements have been found, such as in Estonia (prevalence of tools made of elk and cow bones [10]) and Germany (Falkenwalde–prevalence of tools made of horse bones, cf. Wetzel [13]; Lohberg–prevalence of products made of cow bones, cf. Feustel [14]).

Most Bruszczewo-type tools (approximately two-thirds) were made of the right scapulae, which is likely attributable to the selection of raw materials based on the possibility of adapting them to the preferred arrangement of working edges. This is because both the type of scapulae used (the natural shape of working edges B and C) and technological processes they were subjected to (among others, the way of working the *Spina scapulae* or the surface–the *Facies costalis/*the *Facies lateralis*–on which the blade of working edges A1 and D was formed) were intended to create implements optimised for use by right-handed individuals. These preferences are reflected in the changes in the arrangement of the contact surfaces of working edges A1, A2, B, and C readable between both established morphological types of the Bruszczewo products (cf. Figs 11 and 14). Single cases of deviation in this respect (e.g. tool no. 8) are attributable to these products being used by left-handed individuals.

In the literature, at least 10 theories on the function of the Bruszczewo-type tools have been proposed (cf. Introduction). However, in all cases, these are merely speculations that are not supported by thorough scientific research. Based on the traceological studies we conducted, the use-wear traces on the Bruszczewo-type tools were classified into several types and variants. However, the shared characteristics of these traces are relatively uniform and recurring. Most importantly, for most tools (irrespective of their functional type), the damage visible on the working edges was observed to have a peculiar dualistic nature, manifested in the different characteristics of the traces readable on the blades (the cutting edges) and the parts of the working edges outside this area. The damage seen on the cutting edges is characterised by severe traces of compression and polish with a domed topography and smooth texture that completely obliterates the microrelief of the raw material. Beyond the area of the cutting edge, on the contact surfaces, compression traces are clearly not as well developed (or are completely absent), with the most characteristic element of use-wear damage being an invasive polish with flat topography and heterogeneous and slightly rough texture, related to numerous linear traces that are oriented perpendicularly. These differences most likely arise from the varying traits of the material with which the forementioned parts of the working edge were in contact. The traces readable on the contact surfaces of the tools (beyond the cutting edge’s line) primarily reflect the worked raw materials’ specificity. The characteristics of the damage observed on the cutting edges were also largely affected by the ‘foundation’ on which the work was performed, highly likely (as one may conclude based on the observed damage’s traits) a wooden surface.

Nevertheless, what can be stated regarding the raw material, used for the Bruszczewo-type tools and the activities for which they were employed? The characteristics of the use-wear damage visible on these products suggest that the worked material was somewhat soft yet quite abrasive, as evidenced by the vivid linear traces observed on the tools. In all likelihood, it was also dry (or at least not extremely wet), judging by the absence of a distinct network of osteons on the working edges of the artefacts cf. [90]. Likewise, no traces of burning or heating indicating that the raw material had been subjected to thermal ‘processing’ were observed on them.

Although the experimental studies were extensive and covered numerous activities, they identified only two activities that generated traces of use similar to those visible on the Bruszczewo tools—namely, wheat threshing and pea threshing.

The experimental threshing of bread wheat generated traces analogous to those visible on the Bruszczewo-type tools (especially use-wear type 2 traces). Minor differences between the artefacts and the implements used in the experiments solely comprise the (seemingly) statistically higher abrasiveness of the linear traces readable on the contact surfaces of the working edges of the experimental tools and a slightly higher roughness of the texture of the polish on their non-contact sides. These discrepancies cannot be considered significant, though they may arise from the slightly different biological characteristics of the processed plants, rather than taphonomic changes on the artefacts as a result of post-depositional processes. In Bruszczewo two cereals were cultivated, emmer wheat (*Triticum dicoccum*) and hulled barley (*Hordeum vulgare* var. *vulgare*) [91]. Noteworthily, emmer wheat is a so-called ‘glume’ wheat, which, when threshed, breaks into individual segments (referred to as spikelets) with the grain still tightly enclosed by the chaff (glumes, pales, and lemmas). Therefore, further dehusking is required to release the grain from the chaff. Hulled barley, by contrast, is a ‘free-threshing’ cereal, which responds in a similar way as bread wheat to threshing: its ears break down into chaff (rachis) and grain during threshing [92]. In conclusion, the use-wear traces observed on some of the Bruszczewo-type tools can be linked to the threshing of both emmer wheat and hulled barley.

At first glance, the traces observed on the tool used for experimental threshing of peas differ from most damages observed on Bruszczewo-type tools (especially for use-wear type 1 and 2 traces). However, notably, their general characteristics largely correspond to the type 3 use-wear traces observed on Bruszczewo-type tools, especially those documented by microphotographs captured for tool no. 3 (Fig 12A, 12C and 12E). Additionally, several features typical of use-wear traces from threshing peas were also observed (more or less legible) on Bruszczewo-type tools of other types and functional variants, e.g. greater convexity of the polish topography (cf. Fig 11E and 11H), greater ‘chaoticness’ of the linear traces (cf. Fig 11D), the presence of wide short grooves among the linear traces on the contact side of the working edge (cf. Figs 10A, 10B; 11A; 15C), and the presence of short, perpendicular linear traces on the non-contact sides of the working edges (cf. Figs 14B and 15D). Therefore, some of the analysed tools might have been used also, or even primarily (as is the case with some specimens of use-wear type 3), for threshing peas (or legumes in general). However, the general characteristics of both types of use-wear traces (resulting from threshing cereals and peas) are extremely similar; hence, in the case of tools used for both functions, with one of them clearly dominating, determining this fact in the case of artefact analysis is highly difficult or even impossible.

The suggested link between the Bruszczewo-type tools and cereal and legume processing is also supported by the results of the analysis of the organic residues present on them.

The connection between the plant fibres identified during the first stage of this research and the original function of the analysed Bruszczewo-type tools was considered unlikely. The presence of a mixture of various fibres, likely including flax, cotton and synthetic fibres, does not support the possibility of their archaeological origin. Seemingly, the network of fungal spore hyphae (which have a sticky consistency) possibly trapped fibre particles that entered the artefacts during post-excavation treatment (probably the cleaning and drying of the artefacts during the traceological analysis). Dust particles containing tiny fibres, often of homogenous length, from clothing or other origins could have been the route of entry for the fibre remains identified in association with the Bruszczewo artefacts analysed here. A large portion of the identified fibres probably also originates from cotton towels (and those made of other materials) used to clean tools during the traceological analyses.

In stage two of the residue studies, the analysis primarily involved tools that had not been washed or subjected to traceological analysis; consequently, their surfaces showed no contamination identified during stage one of the study, and the presence of residues was most likely related to the original function of the examined product. These characteristics suggest that the analysed tools may have been involved in the processing of cereals and legumes. The people living at the Bruszczewo site cultivated both of these plants. Large amounts of burnt grain were discovered (emmer wheat *Triticum dicoccon* and barley *Hordeum vulgare*) in the site’s peat part. Legumes (pea *Pisum sativum L*., lentil *Lens Mill*., and likely bitter vetch *Vicia ervilia L*.) dated to the Early Bronze Age are located in the site’s mineral part [91].

The remains of cereals were identified on the surface of some artefacts. These results—in combination with the conclusions of the traceological studies—strongly support the hypothesis that the scapula tools were used for threshing (probably) emmer wheat and barley, the main cereals cultivated in Bruszczewo. Fragments of spongy parenchyma and starch grains attributed to legumes were identified on one artefact. This evidence suggests that at least one of the Bruszczewo scapula tools was used for threshing legumes. However, as indicated by the high analogy of the distinguished use-wear type 3 traces and the damage observed on the experimental tool for threshing peas, more tools were used in this manner, or at least dual-functionally, that is, for both threshing cereals and legumes. Certainly, it cannot be ruled out that similar traces of use will also be generated by the processing (including threshing) of other types of plants. However, such residues were not identified on the working edges of Bruszcewo-type tools; therefore, it is highly probable that these tools can be associated with the processing of cereals and legumes.

Unfortunately, the SEM-EDX analyses failed to add much to the research on the functions of the investigated tools. The most puzzling result herein is the presence of a rare element on the working edges of the artefacts, namely, barium, which has not been found in soil from the site (excluding its post-depositional origin). Nevertheless, it occurred to a high degree in the Bruszczewo ceramics. Perhaps the source of this element is identical in both cases—the clay soil outside the borders of the site—where the raw material for creating pottery was obtained and where the plants were cultivated. Possibly, barium was ‘transferred’ onto the analysed implements as a result of the processing. This issue cannot be resolved without detailed chemical tests of the composition of the soil from the vicinity of the site in Bruszczewo.

As mentioned above, the use-wear damage observed on the analysed tools is relatively uniform and was formed most likely as a result of one type of activity, that is, the threshing of cereals and (less often) legumes. However, certain differences between them have been observed, which serve to distinguish several functional types and their variants among the examined products. As previously noted, some of them arose from using the Bruszczewo-type tools to work with several types/species of plants. Others, could be the result of small differences in how individual artefacts were employed, which also seems to be supported by the fact that use-wear traces of several different functional types have been observed on the working edges of many of the analysed implements.

In the case of Bruszczewo-type tools, traces of functional type 2 should be considered ‘classic’, as they provide a record of ‘unidirectional’ use of the working edges. Some implements of functional type 3 (and perhaps variant 2a) are specimens that could have been employed for working in two directions (with variant 2a most likely providing a record of incipient traces after the orientation of the tool was changed). The parallel linear traces visible on the non-contact sides of the working edges of the implements of functional type 1 (in cases where they are single and the texture of the polish remains smooth) are likely the result of the working edges being wiped (cleaned). However, those on the specimens on which they are multiple and coincide with a linear polish of corrugated topography (including the only tool of type 1a that was found), could have been attributable to the artefacts being used differently, perhaps for processing plants on a hide pad, or even owing to a dual use of these specimens. A fully reliable interpretation of the origin of this use-wear damage requires further experimental studies. Such studies are also required to understand the process of forming the parallel linear traces on the cutting edges of the products classified as functional type 2. Such traces have been identified on the experimental products, but how they were formed remains unclear.

Osseous tools used for threshing are known from only a few prehistoric sites. They were unearthed in Neolithic layers at Grotta dei Piccioni, Italy [93], and in layers dated to this period at Ganj Dareh, Iran [94]. Interestingly, at the latter site, the activity in question was performed using products made of animal scapulae. A similar manner of using tools crafted from these bones was suggested by Anderson for the Abu Hureyra site in Siria [95]. Most recently, this function has been initially attributed to one of the osseous products found in an Early Neolithic grave in southern Poland [96]. Nevertheless, the analysed collection of Bruszczewo-type tools is the first assemblage in such great numbers of products related to this function from one site.

Kroll suggested that in Early Bronze Age Bruszczewo, only ears were collected during harvest [91]. This statement poses some problems of interpretation, as threshing only the ears (without stalks) may be challenging. However, this requires further experimental research. Another solution is that only spikelets were collected, which are the result of threshing hulled cereals like emmer (later subjected to dehusking). This possibility cannot be ruled out either because the data source for Kroll’s statement is uncertain.

## Conclusions

This paper presents the results of technological and functional analyses of Europe’s largest collection of Early Bronze Age scapulae notched tools; thus, it can serve as a starting point for similar studies on products of this form from other sites on the continent. As stressed in the Introduction, this could resolve issues concerning the purpose of these objects, which archaeologists have been preoccupied with for over a century. The analysed Bruszczewo-type tools proved to be an extremely important collection because they provided the first record of threshing cereals and legumes in the central part of Europe during the Early Bronze Age.

The question contained in the title of this article, ‘problem solved?’—which refers to the research on the possible function of the discussed type of tools—was posed in reference to a study by Northe [9], who titled his 2001 article, which provides a summary of previous research on these products, ‘Notched implements made of scapulae–still a problem’. Having presented the findings of studies of the Bruszczewo artefacts, can we finally answer this question positively? It seems that we can, albeit only in part and solely in the context of the Bruszczewo materials. The degree of functional homogeneity of implements classified as the morphological form in question can be determined only after verifying the results presented herein by analysing collections from other sites, particularly those of a different chronology. Nevertheless, even at this point, we will most likely observe some diversity in how these products were used, as suggesting the purpose of cereal threshing for Early Holocene tools, such as those from the Stanovoye 4, site in Russia, is difficult [29].

Likewise, research on the Bruszczewo collection cannot be deemed complete. Further analyses of the residue must be conducted to statistically confirm the occurrence of cereal and legume remains on their working edges. Should legume residues be found on a higher number of tools, and should it appear that the connection between Bruszczewo-type tools and legume processing is strong (as in the case of cereals), it will be necessary to plan a new experimental programme to test other species of these plants and find the one that was most probably processed in Bruszczewo.

## Supporting information

S1 Table Catalogue of artefacts subjected to traceological analysis. (DOCX)
