# Supplementary material for: Notched implements made of scapulae (Bruszczewo-type tools)—A problem solved? Discovering cereal- and legume-threshing techniques in Early Bronze Age Europe through traceological analysis and residue studies
Source: PLoS One. 2024 Sep 13;19(9):e0308700. doi: 10.1371/journal.pone.0308700 (PMC11398652; doi:10.1371/journal.pone.0308700)
Supplement: S1 Table — (DOCX) [file pone.0308700.s001.docx]

Table 1. Catalogue of artefacts subjected to traceological analysis (supplementary material)

| No. | Inv no. | Species;  Right/left shoulder blade (R/L) | Type of tool | Technological traces | Use-wear traces and *residues* | Position in the production chain/  artefact biography | Figure | Comments |
| --- | --- | --- | --- | --- | --- | --- | --- | --- |
| 1 | 3/01 | Cattle  R | I | *Spina scapulae* - clear traces of chipping off bone fragments, then grinded. *Cavitas glenoidalis* removed (in an unspecified way, certainly knapped) and also grinded. The acetabular tubercles and any irregularities were removed (ground). *Margo caudalis* in the cervical part (also the cervix) was removed (cut to the level of the spongy structure and then broken off). ***Method of forming the working edges:*** A1 – ground to *Facies costalis*; A2 – undetermined; B – scraped/ground onto *Facies lateralis*; D – damaged, originally ground ("blade" working on *Facies lateralis*). | **Edge A1:**  - use wear type 1  **Edge A2:**  - use wear type 2  **Edge B, C:**  - morphological type 1, edge B  - use wear type 2: morphological type 1 | U | Fig 2: 1; Fig 10C, F, I;  Fig 13D | - |
| 2 | 3/01 | Cattle  R | - | Production waste, cut-off part of the *Cavitas glenoidalis* neck. Visible traces of longitudinal sawing along the *Margo cervicalis*. Faintly discernible signs of fracturing on the articular surface. | - | P1 | Fig 4: 3 | - |
| 3 | 6124 – 345/04 | Cattle  R? | II | Fragment of the working edge of the tool (edges A and B). ***Method of forming working edges:*** A1 – planed and ground from *Facies costalis/medialis*; A2 – 3: teeth (method of execution unspecified); C – damaged; D – absent. | **Edge A1:**  - use wear type 3 | W | Fig 12A, C, E | - |
| 4 | 41/00 | Unspecified (deer?)  R | II | Specimen partially damaged post-depositionally. The neck part with *Cavitas glenoidalis* was removed. The lateral surface of the specimen was scraped. In the *Facies lateralis*, clear traces remain from the cutting and removal of *Margo cervicalis* (sawing into cancellous mass and then breaking off the removed fragment). In the proximal part, there are no technological traces on *Margo caudalis*; in *Basis scapulae*, the bone was completely removed to the depth of cancellous mass, and the entire surface was ground. **Method of forming working edges:** A1 – scraped and likely planed from *Facies lateralis*; A2 – unspecified; C – ground from *Facies lateralis* when transitioning to edge D; D – similar to edge C. | **Edge A1:**  - use wear type 2  **Edge B, C:**  - morphological type 2, edge C.  **Edge D:**  - variant 2b | U | Fig. 2: 3; Fig 11B, F, J, N;  Fig 15B, F | - |
| 5 | 11187 | Unspecified  - | - | Fragment of a tool, on the surface, only scraping marks are visible. | - | W | - | - |
| 6 | 2133 - 160/04 | Unspecified  - | - | A fragment of the edge A1 (?) has been preserved. On one side, there are visible traces of perpendicular grinding, likely for the creation/repair of the working edge. | **Edge A1:**  - use wear type 1 | W | Fig 10B, E, H | - |
| 7 | 4212 – 257/04 | Cattle  R | I | Fragment of a tool (partially preserved edges A and D). *Spina scapulae* was removed through a combination of flaking and planing. **Method of forming working edges:** A – sawed through the upper part of the bone mass and cancellous mass, then broken off; C ground from *Facies costalis/medialis* (blade on the lateral side). | **Edge D:**  - variant 2b | P3/U | Fig 4: 1; Fig 15D, H, I | The tool illustrates the repair process, i.e. an attempt to create a new working edge on edges A and D. The technological treatments performed remove very well-developed traces of use. Once the damaged working edges were removed, the new edges were likely unused. |
| 8 | 14129 | Wild Boar  R? | II | *Cavitas glenoidalis* fractured. On the *Facies lateralis* of the handle, approximately 1 cm in length, and in the central part of the tool, grinding is visible. Grinding is also evident on one side of the handle, which, with the broken opposite edge, forms a spike. *Margo caudalis medialis* is incised in several places and additionally scraped/polished at the base. *Margo cervicalis* removed (without cutting but simply by breaking off). **Method of forming working edges:** A1 – scraped from *Facies lateralis*; A2 – unspecified; C – unspecified for most of its length, transitioning to edge D, it is ground like D from *Facies lateralis* (cutting edge on *Facies costalis/medialis*). | **Edge A1:**  - variant 2a  **Edge A2:**  - unspecified  **Edge B, C:**  - morphological type 2, Edge C  - variant 1a  **Edge D:**  - unspecified | U | Fig 9B, C, D;  Fig 11D, H, L, O;  Fig 14A, E, L | - |
| 9 | 8177 | Unspecified (Cattle?)  R | I | The specimen is preserved fragmentary (handle of the tool). The articular facet is likely removed through flaking. Irregularities on the *Facies costalis/medialis* of the tool are scraped/ground. *Spina scapulae* is flaked and polished. *Margo caudalis* is removed: sawed and then broken off. On *Facies costalis/medialis*, there are visible traces of three other cuts (probably slips of the blade) that were not continued. On *Margo cervicalis*, clear negatives of three long blades, removed by planing, are evident. **Method of forming working edges:** edge A1 on the preserved fragment scraped longitudinally (traces partially preserved and additionally heavily chamfered by planing/chiselling from the lower side); Edge B – planed from the lower side. | **Edge B, C:**  - morphological type 1, Edge B  - use wear type 2: morphological type 1 | W | Fig 4: 7 | The characteristics of the planing marks indicate the softening of the raw material and the use of a metal knife. |
| 10 | 2027 | Unspecified  L | II | All lateral edges are damaged. Essentially, there are no technological traces, except for remnants of corrections to *Margo caudalis medialis* (unclear technique, visible grinding traces). |  | W | - | The specimen shows signs of fire exposure. |
| 11 | 12156 | Large mammal (cattle?)  R | I | The specimen is preserved fragmentary. The articular facet is removed, probably through flaking (clear individual negatives). A portion of the surface on *Facies costalis/medialis*, in the proximal part, is chiselled out. Upper parts of *Spina scapulae* within the handle area are removed (likely initially flaked, planed, and finally ground). *Margo caudalis* is removed (sawed at an angle and then broken off). **Method of forming working edges:** A1 – scraped from the bottom. | Specimen covered with ferruginous encrustations. | W | Fig 4: 5 | The characteristics of the chiselling marks indicate the use of a metal tool. |
| 12 | 14020 | Large mammal  - | - | Fragment of an object. On two edges, from *Facies costalis/medialis*, there is a visible perpendicular grind. One of them also has a grind from the *Facies lateralis* side. On the third edge, teeth are visible, most likely formed by sawing and planing techniques. | - | - | - | The specimen may be a fragment of another type of tool. |
| 13 | 11019 | Cattle  L | - | Neck part with *Cavitas glenoidalis*. Only traces of its separation from the rest of the scapula are visible, i.e., sawing to the level of the cancellous mass and then breaking off. | - | P1 (W) | Fig 4: 2 | Waste product. |
| 14 | 12183 | Unspecified | - | Fragment of working edges A2 and D. Technological traces are indistinct. | **Edge A2:**  **-** use wear type 2 | W | - | - |
| 15 | 11027 | Unspecified | - | Fragment of the working edge, probably A1. Visible remnants of edge scraping. | Specimen covered with ferruginous encrustations.  **Edge A1:**  - use wear type 1 | W | Fig 16A | - |
| 16 | - | Cattle  R | II | Articular facet removed by incision, breaking, and flaking. *Margo caudalis* was removed, with clear remains of incision on both sides in the proximal part, followed by breaking the edge. **Method of forming working edges:** A1 and probably A2 – processed from the lateral side; C – no processing, transitioning to edge D ground from the lateral side (blade on *Facies costalis/mediali*s); D - ground from *Facies lateralis*, blade on *Facies costalis/medialis.* | **Edge A1:**  - use wear type 3  **Edge A2:**  **-** use wear type 2  **Edge B, C:**  - morphological type 2, edge C  - use wear type 2: morphological type 2  **Edge D:**  - variant 2b | U | Fig 12B, D, F;  Fig 13E, F, G;  Fig 14C, G, J, M;  Fig 15C, G | - |
| 17 | - | Unspecified  - | - | Fragment of a tool, splintered (part near the handle?). Grinding on one of the "poles" of the artefact on *Margo caudalis Facies costalis/medialis* of the scapula. | - | W | - | - |
| 18 | 1031 | No data  R | I | Fragment of the handle of a tool. The articular facet was removed (flaked and partially ground). *Margo caudalis* of the scapula sawn and broken off. *Spina scapulae* flaked, possibly also ground. | - | W | - | - |
| 19 | - | Cattle  L | I | Fragment of the handle of a tool. The articular facet was removed (broken and flaked). *Margo caudalis* of the scapula was removed (sawn from two sides and broken off). *Spina scapulae* flaked, then partially ground. | - | P3? | - | - |
| 20 | 21/01 | Cattle/deer  R | I | Fragmentary form. On *Facies costalis/medialis*, in the neck part, traces of scraping are visible. Traces of this origin are also observed on the *Facies lateralis* of *Margo cervicalis*. **Method of forming working edges:** A – fragmentary, ground from *Facies lateralis*, including *Spina scapulae*, with clear traces of planing; B – probably processed from *Facies lateralis*; part of edge B transitioning to D, and edge D - ground from *Facies costalis/medialis*. | **Edge A1:**  - use wear type 2  **Edge B, C:**  **-** morphological type 1, edge B  - variant 2a  **Edge D:**  - variant 1a | U | Fig 14D, H, K;  Fig 15A, E;  Fig 16B, C, H, I | Edge B in the central part was secondarily planned to create a cavity. It was a repair process, after which the tool was still used. |
| 21 | 6168 - 349/04 | Cattle  L | II | A tool without the preserved part of the handle. **Method of forming working edges:** A1 – planed and ground on *Facies lateralis*; C – no technological traces, only at the transition to edge D, it was ground from *Facies lateralis*; D – poorly preserved. | **Edge A1:**  - use wear type 2  **Edge B, C:**  **-** morphological type 2, edge C  - use wear type 2: morphological type 2  **Edge D:**  **-** variant 2a | U | - | Planing and grinding of edge A was carried out to renew/sharpen it. |
| 22 | F7454 | Horse, Jaw? | II | Specimen with a postdepositionally broken handle. Possible traces of flaking on edge B. **Method of forming working edges:** A1 – ground; A2 – unspecified; C and D – ground along their entire length. | - | U | - | - |
| 23 | 24/96 | Cattle  L | I | Fragment (upper part) of a tool. *Spina scapulae* ground. **Method of forming working edges:** A1 – ground from *Facies costalis/medialis*; A2 – 3 - teeth, method of formation unspecified; B – ground from *Facies costalis/medialis* in the proximal part, transitioning to D ground at a steep angle from *Facies lateralis*, similar to edge D. This way, an edge on the *Facies costalis/medialis* of the tool was created. | **Edge A1:**  - variant 2a  **Edge B, C:**  **-** morphological type 1, edge B  - use wear type 2: morphological type 1 | U | Fig 4: 8 | A poorly preserved, fossilized specimen. |
| 24 | 1362 | Unspecified  - | - | Fragment of a tool, heavily damaged post-depositionally, splintered. On the preserved original surface, there are traces of parallel scraping along the working edge. | **Edge A1:**  - use wear type 2  **Edge B, C:**  **-** unspecified | W | Fig 11C, G, K;  Fig 16D, E, G, H | - |
| 25 | 25/01 | Wild Boar/pig  R | I | Fragment (working part) of a tool. *Spina scapulae* ground; grinding is also visible on the concave lateral planes of *Spina scapulae*. **Method of forming working edges:** A1/A2 – ground from *Facies costalis/medialis*, the method of forming teeth is unclear; B – ground at a steep angle from *Facies lateralis*, transitioning to D ground from *Facies costalis/medialis* similar to edge D. This way, an edge on the *Facies lateralis* of the scapula was created. | **Edge A1:**  - use wear type 1  **Edge B, C:**  - morphological type 1, edge B  - use wear type 2: morphological type 1  **Edge D:**  **-** unspecified.  **Macro and/or micro-residues:** phytoliths associated with the processing of cereal. In addition, starch granules associated with legumes of the *Fabaceae* family and cereals of the *Triticeae* tribe. | U | Fig 4: 10; Fig 9A  Fig 10A, D, G;  Fig 16F;  Fig 17A | Due to the high wear and tear of the tool, it is difficult to say clearly whether we are dealing here only with the A2 edge or also with the A1 edge, which has been partially "chipped" as a result of work and repairs. |
| 26 | 14202 | Red deer  L? | - | Cut-off neck part with an articular facet, a byproduct from tool production. Only traces of circumferential sawing are visible on the specimen, followed by the breaking off of the articular facet. | **Edge B, C:**  - unspecified | W | Fig 4: 4 | - |
| 27 | 14203 | Cattle  R | II | Specimen well-preserved, partially damaged (post-depositionally) working edges. The neck part with the articular facet was sawed off, and the surface of the handle at the cut line of the facet was levelled using scraping/rough grinding. *Margo caudalis* of *Facies costalis/medialis* within the handle scraped, similar to the lateral edge in this area on the lower side of the specimen. *Margo cervicalis* of the scapula was removed (sawn from two sides and broken off). **Method of forming working edges:** A1 – locally on *Facies costalis/medialis*, surface-chiseled to thin the edge, then ground at a sharp angle. The edge from *Facies lateralis* is likely scraped. A2 – damaged; C – transitioning to D, probably planed at a steep angle (as indicated by the wavy surface) and then, together with edge D, ground from *Facies lateralis*, forming an edge on the *Facies costalis/medialis* of the tool. | **Edge A1:**  - variant 2a  **Edge B, C:**  - morphological type 2, edge C  - use wear type 2: morphological type 2  **Edge D:**  - variant 2b | U | - | Metal tools were most likely used in the production of the tool. |
| 28 | - | Unspecified  R | II | Tool formed and preliminarily prepared for use, essentially unused. The articular facet is broken/chipped. *Margo cervicalis* of the scapula removed (technique unspecified, clear remains of flaking in the proximal part). **Method of forming working edges:** A1 - planed from *Facies lateralis*, and then ground. Edge A2 was not (yet?) formed on the tool; C – planed/chiseled from *Facies lateralis* at a much greater angle than edge A. This treatment also includes the "rounded" part of the blade forming its semi-circular shape. In the top part (near edge D), the blade was heavily planed (exposing the cancellous mass), allowing it to smoothly transition into Edge D. The treatment continued from *Facies lateralis*, creating a "blade" on *Facies costalis/medialis*. Edge D - planed from *Facies lateralis* at basically a straight angle – the blade on *Facies costalis/medialis*. | **Edge A1:**  - use wear type 1 | P3 | - | - |
| 29 | 49/99 | Unspecified  L | II | Tool in its original form. *Cavitas glenoidalis* is broken/chipped and, in some places, chiselled. *Margo cervicalis* of the scapula was removed (sawn and broken). Near the cutting line, visible scraping marks on Facies costalis/medialis. *Margo caudalis* of *Facies costalis/medialis* also scraped. No technological traces on edge B. Edge D ground flat. | - | P2/P3 | Fig 3: 6 | Only the D edge has been formed |
| 30 | 11032 | Cattle  L | I | Tool in its original form. *Spina scapulae* is flaked. *Margo caudalis* removed (sawn and broken). Preliminary grinding on the initial break. *Basis scapulae* damaged post-depositionally. *Margo cervicalis* in the proximal part flaked on one side to create a handle. The remaining part is heavily damaged. Method of forming working edges: A – preliminarily ground; B, D - damaged. | **Edge A1:**  - use wear type 1 | P3 | Fig 3: 4 | - |
| 31 | 14467 | Cattle  L | II | The specimen is relatively well-preserved, although it has partially damaged working edges. *Cavitas glenoidalis* is chipped and flaked. *Margo cervicalis* was removed (sawn from two sides and broken). **Method of forming working edges:** A – preserved only in a small fragment in the proximal part, with a delicate grind from *Facies lateralis*; scraping marks along the edge; C – no traces of treatment in the proximal part, in the part near edge D, poorly visible traces of planing; D – damaged. | **Edge A1:**  - use wear type 3  **Edge B, C:**  **-** morphological type 2, edge C | U | - | - |
| 32 | 11663 | Cattle  R | I | Tool in its original form. *Cavitas glenoidalis* chipped, probably flaked. *Spina scapulae* is flaked. *Margo caudalis* of the scapula was removed (sawn from *Facies lateralis*, then broken and ground). **Method of forming working edges:** A1 – scraping in the proximal part from *Facies costalis/medialis*, on the surface; A2 - absent; B – scraping in the proximal part from *Facies costalis/medialis*; transitioning to edge D, grinding from *Facies lateralis*; D - damaged, but similar to B, there is visible grinding from *Facies lateralis.* | - | P3 | Fig 3: 1 | - |
| 33 | 1126 | Unspecified  L | I | *Cavitas glenoidalis* is broken; traces of flaking are evident. *Spina scapulae*: on the handle, clearly flaked; in the working part of the tool, additionally heavily ground. *Margo caudalis* removed (sawn from the upper side and broken). **Method of forming working edges:** A1 – clear traces of longitudinal scraping on *Facies lateralis,* in the proximal part, a well-readable grind continuing to edge B, so that basically the entire *Facies costalis/medialis* of the scapula is ground in this area; A2 – the method of formation is unclear; B – technological traces (aside from the described grinding) are basically unreadable due to heavily developed use-wear. In the vicinity of the ground surface, remnants of point scraping/cutting; D – ground from *Facies lateralis*. | **Edge A1:**  - use wear type 2  **Edge A2:**  - use wear type 2  **Edge B, C:**  - morphological type 1, edge B  - use wear type 2: morphological type 1  **Edge D:**  **-** unspecified | U | Fig 2: 2; Fig 9E-G;  Fig 11A, E, I, M;  Fig 13A, B, C;  Fig 14 B, F, I | - |
| 34 | 3141 | Red deer  R | II | Tool damaged postdepositionally. Cavitas glenoidalis chipped. Margo caudalis delicately planed on Facies lateralis, Margo caudalis on Facies costalis/medialis ground obliquely. Margo cervicalis of the scapula removed (sawn from the upper side and broken). **Method of forming working edges:** A – damaged; C – on *Facies costalis/medialis*, a visible delicate oblique grind, transitioning to D, the edge flaked from *Facies costalis/medialis* and *Facies lateralis*; D – flaked in a similar way to Edge C, locally planed and possibly ground on the edge. | **Edge B, C:**  - morphological type 2, edge C | P3/U | Fig 3: 5 | Edge C is just being formed, but B is already in use and probably being corrected. |
| 35 | 2047 | Horse  - | I? | Fragment of a tool. *Spina scapulae* was flaked and planed, then ground. One of the *Facies lateralis* shaped using sawing and planing techniques. The other one likely at least planed. | - | W | - | - |
| 36 | 11587 | Unspecified  - | I? | Fragment of a tool. *Spina* (?) *scapulae* worked, probably planed. **Method of forming working edges:** a preserved small fragment of the working edge (B?); possible delicate grind on *Facies costalis/medialis.* | - | W | - | - |
| 37 | 3158 | Cattle  R | I | Tool in its original form. *Cavitas glenoidalis* chipped, likely flaked (traces covered by use wear). *Spina scapulae:* flaked on the handle, additionally planed/chiselled within the working part. *Margo caudalis* of the scapula removed (sawn from the upper side and broken). **Method of forming working edges:** A1 – damaged; A2 – absent; B – no processing in the proximal part, in the distal part, near *Basis scapulae* (D) planed from *Facies lateralis*; D – flaked/broken. | **Edge B, C:**  - morphological type 1, edge B | P3/U | Fig 3: 3 | Edge C is just being formed, but B is already in use and probably being corrected. |
| 38 | 62/99 | Wild Boar  R | II | Tool fairly well-preserved, with a chipped handle and partially damaged working edges. Technological traces largely covered by use wear. **Method of forming working edges:** A1 – clearly visible small fragment, processing (likely planing) forming an edge on *Facies lateralis*; A2 – method of formation unclear; C – in the distal part, transitioning to Edge D, planed, probably also ground from *Facies lateralis*; D – ground from *Facies lateralis*. | **Edge A1:**  **-** variant 2a  **Edge A2:**  **-** use wear type 2  **Edge B, C:**  - morphological type 2, edge C  **Edge D:**  **-** unspecified | U | Fig 13H | - |
| 39 | 12116 | Large mammal  R? | II | Fragment of a tool. *Margo caudalis* on *Facies costalis/medialis:* flaked.. | - | W | - | - |
| 40 | 8191 | Unspecified  R | I | Fragment of a tool. *Spina scapulae* was grinded. **Method of forming working edges:** A1 and A2 – clear planing and superficial scraping from *Facies costalis/medialis*. The method of distinguishing the notch (not a tooth) on A2 is unclear. B – processed in the proximal part from *Facies lateralis* (unclear technique); in the part near Edge D, ground (?) from *Facies costalis/medialis* (similar to Edge D, mostly damaged postdepositionally). | **Edge A1:**  **-** use wear type 2  **Edge A2:**  **-** unspecified  **Edge B, C:**  - morphological type 1, edge B  - use wear type 2: morphological type 1  **Edge D:**  **-** unspecified | U/W | Fig 4: 7 | - |
| 41 | 11655 | Unspecified L | I | - | **Macro and/or micro-residues:** phytoliths associated with the processing of cereal. In addition, starch granules associated with legumes of the *Fabaceae* family and cereals of the *Triticeae* tribe.  Fragments of spongy parenchyma tissues have also been identified. | U/W | Fig 17B-H | The specimen was subjected to residue analysis and a verifying (general) traceological analysis. |
